# Identifying causal serum protein–cardiometabolic trait relationships using whole genome sequencing

**DOI:** 10.1093/hmg/ddac275

**Published:** 2022-11-09

**Authors:** Grace Png, Raffaele Gerlini, Konstantinos Hatzikotoulas, Andrei Barysenka, N William Rayner, Lucija Klarić, Birgit Rathkolb, Juan A Aguilar-Pimentel, Jan Rozman, Helmut Fuchs, Valerie Gailus-Durner, Emmanouil Tsafantakis, Maria Karaleftheri, George Dedoussis, Claus Pietrzik, James F Wilson, Martin Hrabe de Angelis, Christoph Becker-Pauly, Arthur Gilly, Eleftheria Zeggini

**Affiliations:** Institute of Translational Genomics, Helmholtz Zentrum München – German Research Center for Environmental Health, Neuherberg 85764, Germany; Technical University of Munich (TUM), School of Medicine, Munich 80333, Germany; Institute of Experimental Genetics, German Mouse Clinic, Helmholtz Zentrum München, German Research Center for Environmental Health (GmbH), Neuherberg 85764, Germany; German Center for Diabetes Research (DZD), Neuherberg 40225, Germany; Institute of Translational Genomics, Helmholtz Zentrum München – German Research Center for Environmental Health, Neuherberg 85764, Germany; Institute of Translational Genomics, Helmholtz Zentrum München – German Research Center for Environmental Health, Neuherberg 85764, Germany; Institute of Translational Genomics, Helmholtz Zentrum München – German Research Center for Environmental Health, Neuherberg 85764, Germany; MRC Human Genetics Unit, Institute of Genetics and Cancer, University of Edinburgh, Edinburgh EH8 9QN, UK; Institute of Experimental Genetics, German Mouse Clinic, Helmholtz Zentrum München, German Research Center for Environmental Health (GmbH), Neuherberg 85764, Germany; German Center for Diabetes Research (DZD), Neuherberg 40225, Germany; Institute of Molecular Animal Breeding and Biotechnology, Gene Center, Ludwig-Maximilians University Munich, Munich 80539, Germany; Institute of Experimental Genetics, German Mouse Clinic, Helmholtz Zentrum München, German Research Center for Environmental Health (GmbH), Neuherberg 85764, Germany; Institute of Experimental Genetics, German Mouse Clinic, Helmholtz Zentrum München, German Research Center for Environmental Health (GmbH), Neuherberg 85764, Germany; German Center for Diabetes Research (DZD), Neuherberg 40225, Germany; Institute of Molecular Genetics of the Czech Academy of Sciences, Czech Centre for Phenogenomics, Vestec 25250, Czech Republic; Institute of Experimental Genetics, German Mouse Clinic, Helmholtz Zentrum München, German Research Center for Environmental Health (GmbH), Neuherberg 85764, Germany; Institute of Experimental Genetics, German Mouse Clinic, Helmholtz Zentrum München, German Research Center for Environmental Health (GmbH), Neuherberg 85764, Germany; Anogia Medical Centre, Anogia 74150, Greece; Echinos Medical Centre, Echinos 67300, Greece; Department of Nutrition and Dietetics, School of Health Science and Education, Harokopio University of Athens, Athens 17671, Greece; Institute for Pathobiochemistry, University Medical Center of the Johannes Gutenberg University Mainz, Mainz 55122, Germany; MRC Human Genetics Unit, Institute of Genetics and Cancer, University of Edinburgh, Edinburgh EH8 9QN, UK; Centre for Global Health Research, Usher Institute, University of Edinburgh, Edinburgh EH8 9QN, UK; Institute of Experimental Genetics, German Mouse Clinic, Helmholtz Zentrum München, German Research Center for Environmental Health (GmbH), Neuherberg 85764, Germany; German Center for Diabetes Research (DZD), Neuherberg 40225, Germany; Chair of Experimental Genetics, TUM School of Life Sciences, Technical University of Munich, Freising 80333, Germany; Institute of Biochemistry, Unit for Degradomics of the Protease Web, University of Kiel, Kiel 24118, Germany; Institute of Translational Genomics, Helmholtz Zentrum München – German Research Center for Environmental Health, Neuherberg 85764, Germany; Institute of Translational Genomics, Helmholtz Zentrum München – German Research Center for Environmental Health, Neuherberg 85764, Germany; Technical University of Munich (TUM) and Klinikum Rechts der Isar, TUM School of Medicine, Munich 80333, Germany

## Abstract

Cardiometabolic diseases, such as type 2 diabetes and cardiovascular disease, have a high public health burden. Understanding the genetically determined regulation of proteins that are dysregulated in disease can help to dissect the complex biology underpinning them. Here, we perform a protein quantitative trait locus (pQTL) analysis of 248 serum proteins relevant to cardiometabolic processes in 2893 individuals. Meta-analyzing whole-genome sequencing (WGS) data from two Greek cohorts, MANOLIS (*n* = 1356; 22.5× WGS) and Pomak (*n* = 1537; 18.4× WGS), we detect 301 independently associated pQTL variants for 170 proteins, including 12 rare variants (minor allele frequency < 1%). We additionally find 15 pQTL variants that are rare in non-Finnish European populations but have drifted up in the frequency in the discovery cohorts here. We identify proteins causally associated with cardiometabolic traits, including *Mep1b* for high-density lipoprotein (HDL) levels, and describe a knock-out (KO) *Mep1b* mouse model. Our findings furnish insights into the genetic architecture of the serum proteome, identify new protein–disease relationships and demonstrate the importance of isolated populations in pQTL analysis.

## Introduction

Cardiovascular and metabolic disorders, such as hypertension, hyperlipidaemia, coronary artery disease (CAD) and type 2 diabetes (T2D), impose a heavy and increasing health burden (1,2). Significant progress has been made in disentangling the complex and overlapping genetic aetiology of these diseases through genome-wide association studies (GWAS), which have successfully identified multiple genetic variants associated with disease risk. At the same time, multiplex proteomic assays have enabled the identification of disease-associated proteins (3–5).

However, statistical association with disease does not always mean that the gene or protein plays a causal role. This can be elucidated by coupling genetics with proteomics to identify genetic variants associated with protein levels, known as protein quantitative trait loci (pQTLs). By complementing pQTL analysis with causal inference approaches such as two-sample Mendelian randomization (MR), non-spurious protein–disease relationships and, therefore, disease pathways, genetic variants, and proteins of clinical relevance can be identified (6–12).

We have previously (10) assessed the genetic architecture of 257 serum protein levels in a Greek isolated cohort, MANOLIS, through which we found 164 independently associated pQTLs for 109 proteins, and demonstrated the value of genetically predicted protein levels in clinical risk models. Here, we substantially increase power by doubling the sample size, meta-analyzing whole genome sequencing data from MANOLIS with an additional isolated population cohort, Pomak. We find 301 independent pQTLs for 170 proteins and describe pQTLs that are driven up in frequency in either discovery cohort, illustrating the value of population isolates in the discovery of protein-associated variation. We further highlight previously undetected causal protein–disease associations using genetic colocalization analysis and two-sample MR.

## Results

### Genetic architecture of 170 proteins

We detect 301 independently associated pQTLs (*P* < 7.45 × 10^−11^) for 170 proteins (Supplementary Material, Table S2) that are present in both cohorts with a consistent direction of effect. Of these, 133 variants belong to loci that were not detected previously in MANOLIS only (10). All protein targets had between one and eight independently associated variants (Supplementary Material, Fig. S1), highlighting the varying complexity of protein level genetic architecture. Additional evidence for replication was sought in a protein level dataset of plasma samples obtained from up to 950 individuals (Methods) from the ORCADES study (13), an isolated population from the Orkney islands in the Northern Isles of Scotland. In sum, 177 (58.8%) pQTLs replicated (Methods) in this independent cohort (Supplementary Material, Table S2).

**Figure 1:**
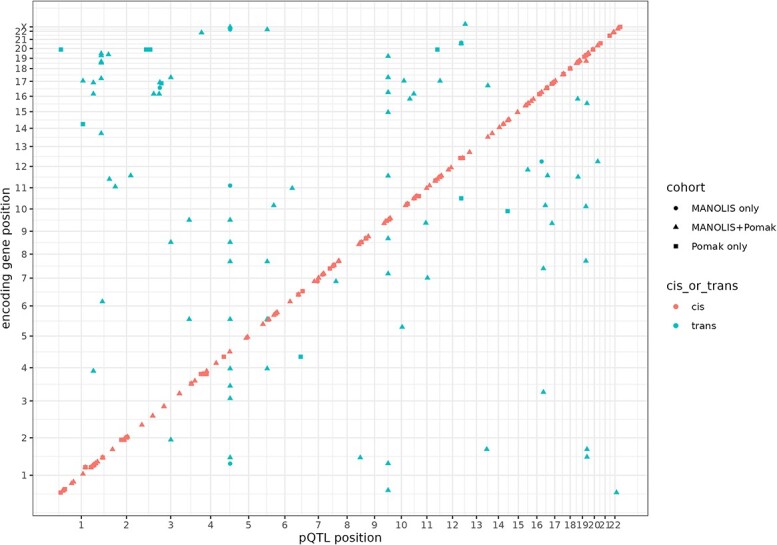
Chromosomal location of *cis*- (red) and *trans*-pQTLs (blue) plotted against the chromosomal location of the gene encoding the proteins of interest. Cis-pQTLs were defined as variants lying within 1 Mb of the start of the gene encoding the target protein.

Detected pQTLs were categorized into *cis-* and *trans*-pQTLs according to their distance to the target protein-encoding gene (Methods); we found 215 *cis-*acting pQTLs for 138 proteins, and 86 *trans-*pQTLs for 63 proteins (Fig. 1). In sum, 31 proteins had both *cis-* and *trans-*pQTLs. By mapping *trans-*pQTLs to their nearest gene, we determined 42 *trans*-pQTLs located in known pleiotropic genes; namely, *ABO, CFH, HLA, F12, FUT2, ST3GAL6* and *KLKB1*. Four of these genes (*ABO, FUT2, F12, KLKB1*) are involved in blood coagulation pathways, whereas *CFH* and *HLA* are closely related to inflammatory response.

Protein QTLs that act in *trans* are also useful for identifying unknown molecular interactions. As proof of principle, we detect an intronic *trans*-pQTL for C-C motif chemokine ligand 3 (CCL3) located within the encoding gene for C-C motif chemokine receptor 3, *CCR3*. CCL3 is a known agonist of CCR3 that may contribute to the aggregation of eosinophils to inflammation sites (14). Mapping *trans*-pQTLs to their causal genes, however, remains a challenge as causal genes are often not the closest ones (8,12) (Supplementary Material, Note 1).

The majority of pQTLs are common variants (minor allele frequency [MAF] > 5%). We find 12 rare (MAF < 1%) pQTLs and 42 low-frequency pQTLs (1% < MAF < 5%). Using Ensembl’s variant effect predictor (VEP), we find altogether 36 (12%) pQTLs that have a most severe consequence of missense, whereas two variants for PRSS27 (*trans*-pQTL) and IL17D (*cis*-pQTL), respectively, are stop-gain variants. The PRSS27-associated variant acts in *trans* and is located within the pleiotropic gene, *FUT2*. The *cis*-pQTL for IL17D and five other missense variants are all rare and were previously undetected in MANOLIS, showing how larger sample sizes provide increased power to detect rare associated variants of severe consequences.

Excluding *trans*-pQTLs located within pleiotropic genes, we find 35 pQTLs (11.6%) in regions that have not been reported in other large-scale pQTL analyses (Supplementary Material, Table S3), comprising 22 *cis*-pQTLs for 18 proteins, and 13 *trans*-pQTLs for 12 proteins. As isolated populations often contain private, rare variants that have drifted up in frequency because of founder effects (15), we additionally interrogate 69 pQTLs that are present in only one discovery cohort, of which 7 replicate in ORCADES (10%) and 28 (40.5%) have not been previously reported (Supplementary Material, Table S2). In sum, 15 novel pQTLs are rare (MAF < 1%) in non-Finnish Europeans (gnomAD) but have drifted up in frequency in one or both of our discovery cohorts by at least 2.25-fold (Table 1; Supplementary Material, Fig. S2; Supplementary Material, Table S4), including four missense variants. None of the 15 variants were present in the replication cohort, and proxies in linkage disequilibrium (LD) failed to replicate. In particular, a *cis-*pQTL for 72 kDa type IV collagenase (MMP2; rs144755357) that has drifted up 95-fold in Pomak is predicted to be deleterious by SIFT and PolyPhen-2 (Supplementary Material, Table S5). The MMP2-increasing variant causes a p.Arg495Gln substitution within the hemopexin C domain, which binds the inhibitor TIMP-2 (16) (Supplementary Material, Fig. S3). We therefore demonstrate the importance of including isolated populations in pQTL association studies as they may contribute to high-impact variants otherwise undetectable in cosmopolitan populations.

**Table 1:** Novel and previously unreported pQTLs that have drifted up in frequency in MANOLIS and/or Pomak. The gnomAD-NFE MAF column contains the minor allele frequencies (MAF) of each variant in non-Finnish Europeans (NFE) from the Genome Aggregation Database (gnomAD). MAFs (gnomAD and 1000 Genomes) of all other detected variants are reported in Supplementary Material, Table S4. The most severe consequences were obtained using Ensembl’s variant effect predictor (VEP). An expanded table containing the genotype counts, Hardy–Weinberg equilibrium test *P*-values, and the full VEP results are in Supplementary Material, Tables S5A and B. Abbreviations: Chr, chromosome; Pos, position; HELIC, Hellenic isolated cohorts; MAF, minor allele frequency; NFE, non-Finnish Europeans

Protein	Chr	Pos	rsID	Cohorts	*cis*/*trans*	HELIC MAF	gnomAD-NFE MAF	Most severe consequence
SUMF2	7	71973324	rs568788425	MANOLIS	*cis*	0.80%	0.04%	Intron
CD1C	1	158292108	rs201448758	MANOLIS+Pomak	*cis*	1.21%	0.01%	Missense
ENO2	12	6862641	rs184861396	MANOLIS+Pomak	*cis*	0.45%	0.20%	Intron
ITGB7	12	53519700	rs541150953	MANOLIS+Pomak	*cis*	1.53%	0.18%	Intron
ACP6	1	121470180	rs114127018	Pomak	*cis*	0.90%	0.01%	Intergenic
APLP1	19	35871901	rs767668877	Pomak	*cis*	1.00%	0.00%	Missense
CD93	1	3888781	rs912070506	Pomak	*trans*	0.20%	0.01%	Intergenic
CD93	2	207672303	rs942471010	Pomak	*trans*	0.40%	0.01%	Intergenic
CD93	2	227266736	rs1396628045	Pomak	*trans*	0.40%	0.01%	Non-transcript exon
IGFBP7	4	67658568	rs539585543	Pomak	*cis*	0.70%	0.02%	Intron
IL1RL2	2	89009162	rs543843028	Pomak	*cis*	2.00%	0.13%	Intergenic
KYAT1	9	126833282	rs746374838	Pomak	*cis*	0.60%	0.00%	Missense
MMP2	16	55496937	rs144755357	Pomak	*cis*	1.30%	0.01%	Missense
PSGL1	12	97893711	rs185338771	Pomak	*cis*	0.40%	0.00%	Intergenic
VSIG2	11	124706898	rs959226701	Pomak	*cis*	0.60%	0.15%	Intergenic

### Identifying proteins associated with cardiometabolic traits

To identify causal relationships between serum proteins and cardiometabolic traits, we applied two-sample Mendelian randomization and colocalization analysis using GWAS summary statistics of complex traits. We defined cardiometabolic traits as follows: all lipid traits; glycaemic traits; diabetes; kidney disease and measures of kidney function; all heart conditions; hypertension; and body-mass index (BMI) (Methods). We find 43 serum proteins that are associated with at least one cardiometabolic trait (Supplementary Material, Table S6 and S7).

**Figure 2:**
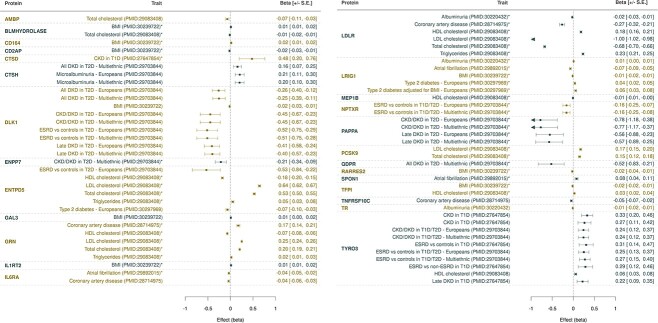
Two-sample Mendelian randomization between proteins (exposure) and cardiometabolic traits (outcome), using only downloaded summary statistics. Points represent the effect size (beta) and direction of each causal association, with errors bars representing ±SE. Arrows indicate beta coefficients that are below −1. Actual beta and SE values are given to the right of each plot. Traits marked with an asterisk (*) indicate that a Wald ratio test was performed; otherwise, the inverse-variance weighted method was used. Full MR results with MRBase traits are given in Supplementary Material, Table S6.

Of these, 18 proteins show strong evidence of causal association (≥2 instrumental variables, using the inverse variance-weighted [IVW] method) with at least one cardiometabolic trait (Fig. 2). Of note are the TYRO3 (tyrosine-protein kinase receptor), DLK1 (protein delta homologue 1) and CTSH (cathepsin H) proteins, which are significantly associated with diabetic kidney disease (DKD). Increased TYRO3 and CTSH levels are associated with an increased risk of DKD in individuals with type 1 or 2 diabetes, and reduced DLK1 levels are associated with an increased risk of DKD in individuals with T2D. Whereas CTSH and DLK1 have not been associated with kidney disease (Supplementary Material, Note 2), studies have shown increased *TYRO3* mRNA expression (17) and increased circulating and urinary TYRO3 levels (18) in patients with DKD, further supporting a causal role. We also note that TYRO3 is targeted by an approved drug for rheumatoid arthritis, fostamatinib, highlighting an opportunity for the repurposing of fostamatinib to treat DKD. We elaborate on other previously unreported examples in Supplementary Material, Note 2.

The MR analysis further validates known protein–disease links, showing causal associations between increased serum LDLR (low-density lipoprotein [LDL] receptor) protein and decreased LDL, total cholesterol and risk of coronary heart disease (19). We also replicate a previously reported finding showing that LRIG1 (leucine-rich repeats and immunoglobulin-like domains 1) lies on the causal path for atrial fibrillation, T2D and self-reported hypercholesterolemia (10).

For two proteins, sulfatase modifying factor 2 (SUMF2; Supplementary Material, Note 2) and meprin A subunit beta (*Mep1b*), we observe association with cardiometabolic traits using novel replicating pQTLs as instrumental variables. We find that decreased serum *Mep1b* is causally associated with increased HDL levels (Wald ratio *P*_FDR_ = 3.38 × 10^−2^; beta = −0.008; SE = 0.002). The intronic *cis-*pQTL, rs680321, is robustly associated with serum *Mep1b* (MAF = 0.37; beta = −1.07; SE = 0.026; *P* = 2.50 × 10^−372^; Supplementary Material, Note 3). Two other independently associated *Mep1b cis*-pQTLs are private to Pomak (rs763953724, rs1410442909); both variants are non-existent in non-Finnish Europeans and lie upstream of the *Mep1b* gene.

To better understand the potential metabolic role played by *Mep1b*, we systematically phenotyped an existing *Mep1b* KO mouse model at the German Mouse Clinic. Monitoring body weight from age 9 to 19 weeks revealed that *Mep1b* depletion in the mouse impacts on the body mass of females, which were heavier as a result of increased adiposity (Supplementary Material, Note 4; Supplementary Material, Figs S4–S6; Supplementary Material, Table S8). This sex-specific effect was not observed for the *cis*-pQTL, rs680321 (sex heterogeneity *P* = 0.086; Supplementary Material, Fig. S7 and Supplementary Material, Table S9).

## Discussion

The relationship between *Mep1b* and cholesterol or adiposity remains largely unexplored. *Mep1b* is a metalloprotease that is involved in post-translational proteolysis of numerous targets (20,21) in mammals. Closely related to meprin α (MEP1A), both proteins have been implicated in inflammatory disorders, Alzheimer’s disease, kidney disease and cancer (20). Several substrates of *Mep1b* have also been linked to cholesterol levels, such as dipeptidyl peptidase 4 (DPP4) and amyloid precursor protein (APP) (22,23). Results from our MR analysis and mouse phenotyping support a direct role of *Mep1b* in influencing adiposity, which is a risk factor for a multitude of complex diseases, including those previously linked to *Mep1b*. Given its involvement in complex networks, however, further experiments will be needed to identify specific pathways.

Our causal inference analysis additionally revealed cardiometabolic traits that are associated with multiple shared proteins (Supplementary Material, Figs S8–S10). LDL cholesterol, total cholesterol and triglyceride levels were all causally associated with the serum levels of seven proteins: GRN, LDLR, SUMF2, KIM1, ENTPD5, CHI3L1 and FGF21. HDL cholesterol was associated with four of the same proteins (GRN, LDLR, SUMF2, ENTPD5), but additionally with eight other proteins (TYRO3, HBEGF, SPON1, SCF, TIMP4, TFPI, MEP1B, ANGPTL1, AXL) that were not significantly associated with LDL or total cholesterol, suggesting a complex and distinct underlying proteomic landscape. This demonstrates the potential of such analyses to furnish insights into molecular similarities and differences between similarly presenting diseases or disease subtypes in future studies, facilitating efforts for more precise diagnosis and treatment.

In this work, we detect 133 new pQTLs, 40% of which are *trans*-pQTLs for 48 proteins, including the *CCR3-*CCL3 receptor-ligand interaction. We were able to reproduce 92% of the 164 independent pQTLs reported previously (10), including 12 variants exclusive to MANOLIS. The remaining 13 pQTLs (12 *cis*, 1 *trans*) were not reproduced because of either the exclusion of the protein from meta-analysis (QC failure) or a loss of significance. Overall, 59% of our pQTLs replicated in an independent cohort. There are several possible explanations for lack of replication, including insufficient statistical power because of the smaller sample size of the replication cohorts, a lack of proxies for private variants, and differences in cell type and protein composition between serum (MANOLIS and Pomak) and plasma (ORCADES) (Supplementary Material, Fig. S11).

Population isolates have special population genetics characteristics that can boost the discovery of rare variant associations. Here, we identify 15 rare pQTLs that have drifted up in frequency in one or both cohorts. Whole genome sequencing enables access to the analysis of rare variants through gene-based burden testing. We have recently described (24) five rare variant burden pQTLs in MANOLIS, Pomak and ORCADES that are independent of the single point signals reported in this work. Projects with larger sample sizes will further increase power and are currently underway.

We recognize several limitations to this work. First, as Olink’s immunoassay relies on the binding of antibodies to target antigens, genetic variation can alter binding sites and, therefore, the affinity of the antibody probes to the target protein. This may result in association signals that reflect altered protein structure rather than changes in protein abundance. For 25 proteins with protein-altering variants (based on Ensembl VEP classification [Methods]), we checked for such effects through a comparison of proteomic data by Olink versus an aptamer-based assay by Somalogic (with different antigen binding sites) in an independent cohort, Fenland (12). We observed good correlation (Spearman correlation>0.5) for 13 (59%) of 22 proteins that were measured using both technologies (Supplementary Material, Table S10), suggesting genuine pQTL signals. Other than altered antibody binding as a result of protein structure changes, weak correlations may be explained by different technical and protein characteristics, as recently investigated (25). Orthogonal validation is therefore necessary for accurate downstream biological interpretation.

Secondly, the validity of the two-sample MR results relies on the assumptions that the genetic instruments (pQTLs) influence the outcome (cardiometabolic trait) only through the exposure (protein level) and are not associated with confounders (Methods). Moreover, we note that the GWAS summary statistics used in this analysis were not derived from WGS-based studies, and therefore several of our instruments were not found in these datasets and could not be used. As we only assess causality unidirectionally, future studies will benefit from bidirectional analyses using larger, sequence-based exposure and outcome GWAS datasets that can produce a greater number of reliable instruments and provide validation. Finally, all individuals in the discovery and replication cohorts are of European descent. Larger, ethnically diverse sample sizes are needed to fully characterize the genetic architecture of the serum proteome.

## Materials and Methods

### Sequencing and variant calling

The two cohorts were sequenced in an identical way. Genomic DNA (500 ng) from 1482 and 1642 samples for MANOLIS and Pomak, respectively, was subjected to standard Illumina paired-end DNA library construction. Adapter-ligated libraries were amplified by six cycles of PCR and subjected to DNA sequencing using the HiSeqX platform (Illumina) according to manufacturer’s instructions.

Basecall files for each lane were transformed into unmapped BAMs using Illumina2BAM, marking adaptor contamination and decoding barcodes for removal into BAM tags. PhiX control reads were mapped using BWA Backtrack and were used to remove spatial artefacts. Reads were converted to FASTQ and aligned using BWA MEM 0.7.8 to the hg38 reference (GRCh38) with decoys (HS38DH). The alignment was then merged into the master sample BAM file using Illumina2BAM MergeAlign. PCR and optical duplicates are marked using biobambam markduplicates and the files were archived in CRAM format.

Per-lane CRAMs were retrieved and reads pooled on a per-sample basis across all lanes to produce library CRAMs; these were each divided in 200 chunks for parallelism. GVCFs were generated using HaplotypeCaller v.3.5 from the Genome Analysis Toolkit (GATK) (26) for each chunk. All chunks were then merged at sample level, samples were then further combined in batches of 150 samples using GATK CombineGVCFs v.3.5. Variant calling was then performed on each batch using GATK GenotypeGVCFs v.3.5. The resulting variant callsets were then merged across all batches into a cohort-wide VCF file using bcftools concat.

### Variant and sample quality control

Variant-level QC was performed using the Variant Quality Score Recalibration tool from the GATK v. 3.5–0-g36282e4 (26), using a tranche threshold of 99.4% for SNPs, which provided an estimate false positive rate of 6% and a true positive rate of 95%. For INDELs, we used the recommended threshold of 1%. For sample-level QC, we made extensive use of genotyping array datasets in overlapping samples, which provided sample matching information for 1386 and 1511 samples in MANOLIS and Pomak, respectively. In MANOLIS, a total of 25 individuals were excluded (*n* = 1457) based on sex checks, low concordance (<0.8) with chip data, duplicate checks, average depth (<10×), missingness (>0.5%) and contamination (Freemix or CHIPMIX score from the verifyBamID suite^32^ > 5%). This number was 27 for the Pomak cohort. In the case of sample duplicates, the sample with highest quality metrics (depth, freemix and chipmix score) was kept.

### Proteomics

The serum levels of 275 unique from three Olink (https://www.olink.com/) panels—Cardiovascular II, Cardiovascular III and Metabolism—were measured using Olink’s proximity extension assay (PEA) technology (Supplementary Material, Table S1). Briefly, for each assay, the binding of a unique pair of oligonucleotide-labelled antibody probes to the protein of interest results in the hybridization of the complementary oligonucleotides, which triggers extension by DNA polymerase. DNA barcodes unique to each protein are then amplified and quantified using microfluidic real-time qPCR. Measurements were given in a natural logarithmic scale in Normalized Protein eXpression (NPX) levels, a relative quantification unit. NPX is derived by first adjusting the qPCR Ct values by an extension control, followed by an inter-plate control and a correction factor predetermined by a negative control signal. This is followed by intensity normalization, where values for each assay are centred around its median across plates to adjust for inter-plate technical variation. Further details on the internal and external controls used can be found at http://www.olink.com. Additionally, a lower limit of detection (LOD) value is determined for each protein based on the negative control signal plus three standard deviations. In this study, NPX values that fall below the LOD were set to missing.

We adjusted all phenotypes using a linear regression for age, age squared, sex, plate number and per-sample mean NPX value across all assays, followed by inverse-normal transformation of the residuals. We also adjusted for the season, given the observed annual variability of some circulating protein levels. Given the dry Mediterranean climate of Crete, we define the season of collection as hot summer or mild winter. Plate effects are partially offset by the median-centring implemented by Olink. MANOLIS and Pomak samples were plated in the order of sample collection, which results in plate and season information to be largely correlated.

In MANOLIS, we excluded 13 protein measurements across all panels with missingness or below-LOD proportion greater than 40%. BNP was measured across all three panels and was excluded because of high missingness in all three. In sum, 26, 2 and 14 samples failed vendor QC and were excluded from Cardiovascular II, III and Metabolism, respectively. Also, 42 samples were excluded because of missing age. In Pomak, we excluded 15 proteins and 49, 6 and 13 samples in Cardiovascular II, III and Metabolism. No samples were excluded because of missing covariates. Seven proteins in MANOLIS and five in Pomak were further excluded because of failing QC in the other cohort. A total of 255 proteins were included in the final single-point analysis (Supplementary Material, Table S1).

### Single-point association and meta-analysis

We carry out single-point association using the linear mixed model implemented in GEMMA v.0.94 (27). We use an empirical relatedness matrix calculated on an LD-pruned set of low-frequency and common variants (MAF > 1%) that pass the Hardy–Weinberg equilibrium test (*P* < 1 × 10^−5^). We further filter out variants with missingness higher than 1% and MAC < 10. Following single-point association, a further seven proteins (GDF15, TFF3, TINAGL1, LOX1, SRC, CTSL1, IDUA) were excluded because of having a genomic control λ_GC_ < 0.97 or λ_GC_ > 1.05 after association in either cohort.

GEMMA truncates alleles to a single character. In order to enable unambiguous meta-analysis of indels, we updated alleles in summary statistics by matching it to the VCF. More precisely, we join both files by chromosome and position, and match the alleles by frequency for biallelics. For multiallelics, we compute the difference in allele frequency between the GEMMA output MAF, which is based on samples with non-missing phenotypes, and the AF fields of each allele in the VCF, and use the alleles with the lowest difference.

We use the 25 March 2011 release of METAL (28) for meta-analysis of 248 proteins using inverse-variant based weighting. Full summary statistics are available for download from the GWAS Catalogue (https://www.ebi.ac.uk/gwas/); accession IDs are provided in Supplementary Table 14.

### Signal extraction and conditional analysis

Using a *P*-value threshold of 1 × 10^−6^, 495 signals were extracted using the peakit.py routine of PeakPlotter commit 545191d6db51d87f2b549351e5cda19aaf50330e (https://github.com/hmgu-itg/peakplotter), after filtering out index variants with a minor allele count (MAC) of <10 or do not pass the Hardy–Weinberg equilibrium test. PeakPlotter is based on a combination of distance-based and LD-based pruning; specifically, the software sorts variants passing the significance threshold by increasing the *P*-value, then for each variant, computes SNPs in LD greater than *r*^2^ = 0.2, removes them and moves on to the next variant. Variants selected in this way located within <2 Mb of each other are then grouped together, and the index variant is set to the variant with the lowest *P*-value. Each index variant defines a signal, and we use locus and signal interchangeably in this article. A total of 380 index variants passing the study-wide significance threshold of *P* < 7.45 × 10^−11^ were extracted. We then extracted independent SNV at each associated locus using an approximate conditional and joint stepwise model selection analysis as implemented in GCTA-COJO^34^, using merged cross-cohort genotypes for LD calculation. To avoid overfitting when too many predictors are included in the model, we perform LD-based clumping using Plink v.1.9 (29) (www.cog-genomics.org/plink/1.9/), based on an *r*^2^ value of 0.1 and a window of 1 Mb before the GCTA-COJO analysis (30). The extended LD present within population isolates can cause very large peaks to be broken up into several signals. We identified and manually investigated 44 regions where multiple peaks were present in close proximity of each other, reducing the number of independent signals to 257 and the number of conditionally independent variants to 370 (301 present in both cohorts).

### Sex-specific meta-analysis

To look for sex-specific pQTLs, we investigated the heterogeneity between males and females for all 370 conditionally independent pQTLs present in at least one cohort. Single-point association analyses for males and females in both discovery cohorts were first run separately for each pQTL using GEMMA v.0.94 (27), using the same methods as described for the main single point analysis. With the output files, we then performed a sex-specific meta-analysis using the GWAMA v2.2.2 software (31,32) by specifying the —sex option. None of the 370 pQTLs show significant sex heterogeneity using a Bonferroni-corrected *P*-value significance threshold (*P* < 1.35 × 10^−4^) (Supplementary Material, Table S9).

### Defining *cis*- and *trans*-pQTLs

We define *cis-*pQTLs as variants that lie within 1 Mb upstream or downstream of the encoding gene, whereas *trans*-pQTLs are all variants lying outside of this region.

### Comparison of Olink and Somalogic proteomic data in Fenland

*Cis-*acting protein-altering variants may result in false-positive associations because of epitope effects. We note that 26 *cis-*acting variants for 25 proteins have a potentially protein-truncating effect (IMPACT of MODERATE or HIGH according to Ensembl VEP). Comparison of Olink measurements with an alternative assay, Somalogic, in the Fenland (12,25) cohort (https://www.omicscience.org/apps/pgwas/) showed good correlation between the two measurements for 13 out of 22 proteins (with *cis-*pQTLs) with both Olink and Somalogic proteomic data (Supplementary Material, Table S10).

### Significance threshold

We based our significance threshold on the effective number of variants and traits analyzed. We excluded variants with MAC < 10 from the MANOLIS cohort, then performed LD-pruning using Plink v.1.9 (29) using the parameter—indep 50 5 2. This yielded an *N*_eff_ = 5 078 182 unique variants for MANOLIS. As computing a similar value for the meta-analysis would have required a computationally intensive merging of genotypes across cohorts and handling of cohort-specific variants, we note that the Pomak estimate is similar and that the majority of variants in the meta-analysis will be common to both cohorts, with a further portion of cohort-specific variants likely in LD with common ones. We therefore use the MANOLIS *N*_eff_ in our analysis. For *M*_eff_, the effective number of phenotypes, we compute the ratio of the eigenvalues of the phenotype correlation matrix to its maximum and obtain 132. The resulting *P*-value threshold is 7.45 × 10^−11^.

### Replication

Replication was performed in the ORCADES isolated cohort from the Orkney archipelago in the Northern Isles of Scotland (13). In sum, 1348 samples were sequenced using the same WGS protocol as described for MANOLIS and Pomak. An identical phenotype transformation was performed on 275 proteins from the CVDII, III and META Olink panels in 995 samples. Because of quality control, between 928 and 950 samples overlapped between the WGS and Olink datasets. All 255 proteins analyzed in MANOLIS and Pomak were also found in the ORCADES dataset. Association was performed using GCTA v.1.93.0 beta using the MLMA algorithm (33). In ORCADES, using common LD-pruned variants for calculating the relatedness matrix was not sufficient, as persistent inflation was present. We assumed this was because of a different relatedness structure being expressed in rare variants, and we therefore included all sequence variants in the relatedness calculation, using five partitions of the autosomal genome. Following this, inflation was controlled. We sought replication for each of the 370 independent variants identified by COJO that are present in at least one cohort, using a Bonferroni threshold of 0.05/371 = 1.35 × 10^−4^.184 variants replicated in this way.

### Novelty

Previous associations with identical proteins was of particular interest as it determines novelty of our findings. To assess whether a protein had been previously studied, we examined protein lists and summary statistics from 33 large published proteomics GWAS (Supplementary Material, Table S3). To determine the novelty of genetic *cis*- and *trans*-association with proteins in our study, we first determined previously reported variants within a 2 Mb window around the association peaks. We used GEMMA (27) to perform association analysis using previously reported independent variants as covariates. The variants were declared novel if either there were no known signals in the 2 Mb window, or the associations were still study-wide significant (*P*-value threshold: 7.45 × 10^−11^) after conditioning. For *trans* associations, we further annotated signals depending on whether they fell within highly pleiotropic genes that were associated with more than 1 protein in the current study and had evidence of additional associations in the literature (*KLKB1, ABO, APOE, FUT2, F12, VTN, CFH, HLA*), or whether they were independent of any *cis* signals in the vicinity. After this procedure, 42 *cis*-associated variants for 30 proteins were either not within 1 Mb or independent of a signal reported in previous proteomics GWAS. In sum, 37 *trans-*associated variants for 34 proteins were both novel and independent from *cis* loci. Only 15 of these were not located within highly pleiotropic genes. For all loci annotated as provisionally novel using the above method, we queried the GWAS Catalogue (34) (https://www.ebi.ac.uk/gwas/home)in a 2 Mb window through the Ensembl (35) REST API, as well as our PhenoScanner results. As proteomics GWAS signals are often designated generically in Ensembl, we additionally performed direct queries to the GWAS catalogue REST API when phenotype descriptions were not specific enough. We manually investigated the list of signals in search of variants associated with the protein trait of interest. When such a variant was found, conditional analysis was performed and the novelty status was updated accordingly. Novelty of each independent variant is annotated in Supplementary Material, Table S2.

### Variant consequences

Consequence was evaluated using Ensembl VEP (35,36) for each variant with respect to any transcript of the *cis* gene for *cis-*associated variants and to the mapped gene for *trans-*associated variants. For *trans* associations, variants were manually mapped to any gene in a 1 Mb window coding for known ligands or interactants when they were not contained within gene boundaries. In sum, 38 replicating independent variants were protein-altering variants with a most severe consequence equal to or more severe than missense (https://www.ensembl.org/info/genome/variation/prediction/predicted_data.html) according to Ensembl VEP. For every variant, we extracted tagging SNVs at *r*^2^ > 0.8 using PLINK; however, none of these tagging variants had a more severe consequence on the target gene than the independent variant. Similarly, we overlapped all independent variants with regulatory features using the Ensembl REST API. 21 variants in 19 loci overlapped with a regulatory feature. Variant consequences are annotated in Supplementary Material, Table S2.

### Gene expression QTL colocalization

We perform colocalization testing with eQTL data from the GTEx database (37) (https://gtexportal.org/home/). First, to account for multiple independent variants at the same locus, for every signal, regions are extended 1 Mb either side of every independent variant, and associations are conditioned on every other variant in the peak using GCTA-COJO; the results are used as input for the colocalization analysis. For *cis* signals, expression information for the *cis* gene is extracted from the GTEx database over the same region. For *trans* signals, expression information is restricted to all genes located within a 2 Mb region surrounding the variant. Then, for every variant/gene pair, we perform colocalization testing using the fast.coloc function from the gtx R package (https://github.com/tobyjohnson/gtx).We use the commonly chosen value of 0.8 as a posterior threshold to declare colocalization (38), and default values of 1 × 10^−4^, with a standard deviation of 1, for the prior probability of a variant to be causal for either trait, and 1 × 10^−5^, with a standard deviation of 1, for the prior probability of a variant to be causal for both traits. In sum, 77 (35%) independent *cis* variants colocalize with an expression quantitative trait locus for the *cis* gene. In addition, we find that 61 (73%) *trans*-pQTL variants colocalize with eQTLs for at least one gene in their vicinity (±1 Mb), in any tissue (Supplementary Material, Table S11; Supplementary Material, Figs S12–S13; Supplementary Material, Note 1).

### PheWAS colocalization

We use the PhenoScanner python command line tool (39,40) (https://github.com/phenoscanner/phenoscannerpy) to query 1 Mb upstream and downstream of every lead variant in each signal. We only considered previous associations with a reported *P*-value of 0 < *P* < 5 × 10^−8^. Using the PhenoScanner associations, we then perform colocalization testing using the same input pQTL data and methods that were used for the eQTL colocalization analysis. We additionally perform colocalization testing using downloaded summary statistics for atrial fibrillation, T2D, Alzheimer’s disease, albuminuria, BMI, waist-hip ratio, estimated glomerular filtration rate, diabetic kidney disease and lipid levels. References to each study and full pheWAS colocalization results are presented in Supplementary Material, Table S7.

### Drug target evaluation

For evaluating whether associated genes were drug targets, we used the OpenTargets (41) and DrugBank (42) databases. We accessed OpenTargets using the OpenTarget API. We converted the DrugBank XML file to flat files using the dbparser R package, and performed gene name matching using the USCS Gene Info database (https://genome.ucsc.edu/), downloaded May 6, 2019.35 of the proteins for which a signal was detected at study-wide significance were targeted by drugs according to OpenTargets. This was true for 70 proteins when queried against the DrugBank database (Supplementary Material, Table S12). In sum, 29 proteins are targeted by drugs according to both OpenTargets and DrugBank databases.

### Mouse phenotype evaluation

We use the Ensembl (35) REST API to extract mouse orthologs for all of the 170 genes that encode proteins for which genetic associations were found in our study. According to the IMPC (43) API (https://www.mousephenotype.org/), KO experiments for 36 of these orthologs were associated with 70 unique phenotypes, with a *P*-value smaller than 1 × 10^−4^ (Supplementary Material, Table S13).

### Two-sample MR

We extracted variants characterized as independent signals by GCTA-COJO (30) on a protein-by-protein basis across all *cis*- and *trans-*loci, and excluded novel variants without an rsID. For each remaining variant, we then extracted their pQTL summary statistics. When a variant was not present in the outcome GWAS summary statistics, we considered pQTL summary statistics for tagging positions with *r*^2^ > 0.8. All such records were then merged by protein and carried over to MR analysis using the MRBase R package (44), where they were merged with the exposure datasets by rsID. MR was performed for 105 proteins on a set of 261 medically relevant traits available in MRBase. We defined cardiometabolic traits as: all lipid traits; glycaemic traits; diabetes; kidney disease and measures of kidney function; all heart conditions; hypertension; and BMI. These are annotated in Supplementary Material, Table S6. As all of our instruments involved a small number of variants (≤10), we used the inverse-variance weighted method, except for single-instrument analyses where we use the Wald ratio test, which consists of dividing the instrument-outcome by the instrument-exposure regression coefficient. All *P*-values were adjusted for multiple testing using the Benjamini–Hochberg method, using the adjusted *P* < 0.05 as the threshold for significant association.

An important caveat of our overlap-maximizing approach is that we did not require overlapping variants to be lead variants in the outcome trait GWAS. This could potentially lead to false-positives for single-instrument tests if the variant is located at the shoulders of an association peak in the outcome trait GWAS. The future availability of population-scale association studies with WGS or WES will greatly enhance the variant overlap compared with GWAS, and hence increase the power of MR analyses in proteomics. In addition to summary statistics available in MRBase, we also leveraged summary statistics manually downloaded from recent large association studies for: albuminuria, diabetic kidney disease, atrial fibrillation, BMI, CAD, lipid levels, T2D. PMID references for these studies are provided in Supplementary Material, Table S6.

### *Mep1b* mouse model

*Mep1b* −/− (C57BL/6 N) mouse model is described in our previous study (45). The targeted mutation leads to the disruption of the catalytic centre in exon7 of the wild-type allele.

### Mouse phenotyping

Mice were maintained in IVC cages with water and standard mouse chow according to the directive 2010/63/EU, German laws and GMC housing conditions (https://www.mouseclinic.de). All tests were approved by the responsible authority of the district government of Upper Bavaria.

In total, 18 mutant mice (9 males, 9 females) and wild-type control littermates (10 males, 10 females) underwent a systematic, comprehensive phenotyping screen by the German Mouse Clinic at the Helmholtz Zentrum Muenchen (https://www.mouseclinic.de) as previously described (46–49). This screen started at the age of 8 and 9 weeks for male and females respectively and covered multiple parameters in the areas of behaviour, cardiovascular function, clinical chemistry, dysmorphology, energy metabolism, eye analysis and vision, haematology, immunology, neurology, allergy and pathology.

### Body weight

Body weight was measured at different time-points at a range of 8–19 weeks.

### Body composition analysis

Body composition was analyzed at 13 and 18 weeks. Lean tissue and body fat in live mice without anaesthesia were measured by the whole-body composition analyzer (Bruker MiniSpec LF 50) based on Time Domain Nuclear Magnetic Resonance.

### Blood collection

Blood samples were collected under isoflurane anaesthesia by retrobulbar puncture after overnight food withdrawal at 11–12 weeks of age and as a final blood withdrawal from ad libitum fed animals at 19–20 weeks. Blood samples for clinical chemistry analyses were collected in Li-heparin-coated tubes and stored at room temperature for one to three hours until centrifugation (4500 × g, 10 min) and separation of plasma aliquots for further analyses.

### Clinical chemistry

The clinical chemistry analyses of circulating biochemical parameters in blood was performed using a clinical chemistry analyzer (AU480 autoanalyzer, Beckman Coulter, Krefeld, Germany). Fasting plasma lipid and glucose levels at 11–12 weeks of age and a broad set of parameters from fed animals at 19–20 weeks were measured using the respective kits provided by Beckman Coulter, including various enzyme activities as well as plasma concentrations of specific substrates and electrolytes in *ad libitum* fed mice (50).

### Statistics

Data generated by the German Mouse Clinic were analyzed using R (Version 3.2.3). Tests for genotype effects were made by Wilcoxon rank sum test, linear models, or ANOVA depending on the assumed distribution of the parameter and the questions addressed to the data. A *P*-value <0.05 has been used as level of significance; a correction for multiple testing has not been performed. Figures were prepared using GraphPad Prism version 7.00 for Windows (GraphPad Software, La Jolla, California, USA).

## Supplementary Material

GP_Supplementary-figures_ddac275

Supplementary_Table_1_ddac275

Supplementary_Table_2_ddac275

Supplementary_Table_3_ddac275

Supplementary_Table_4_ddac275

Supplementary_Table_5_ddac275

Supplementary_Table_6_ddac275

Supplementary_Table_7_ddac275

Supplementary_Table_8_ddac275

Supplementary_Table_9_ddac275

Supplementary_Table_10_ddac275

Supplementary_Table_11_ddac275

Supplementary_Table_12_ddac275

Supplementary_Table_13_ddac275

Supplementary_Table_14_ddac275

GP_Supplementary-text_ddac275
