# Supplementary material for: Identifying causal serum protein–cardiometabolic trait relationships using whole genome sequencing
Source: Hum Mol Genet. 2022 Nov 9;32(8):1266–75. doi: 10.1093/hmg/ddac275 (PMC10077504; doi:10.1093/hmg/ddac275)
Supplement: GP_Supplementary-figures_ddac275 [file gp_supplementary-figures_ddac275.pdf]

Supplementary information

Supplementary figures

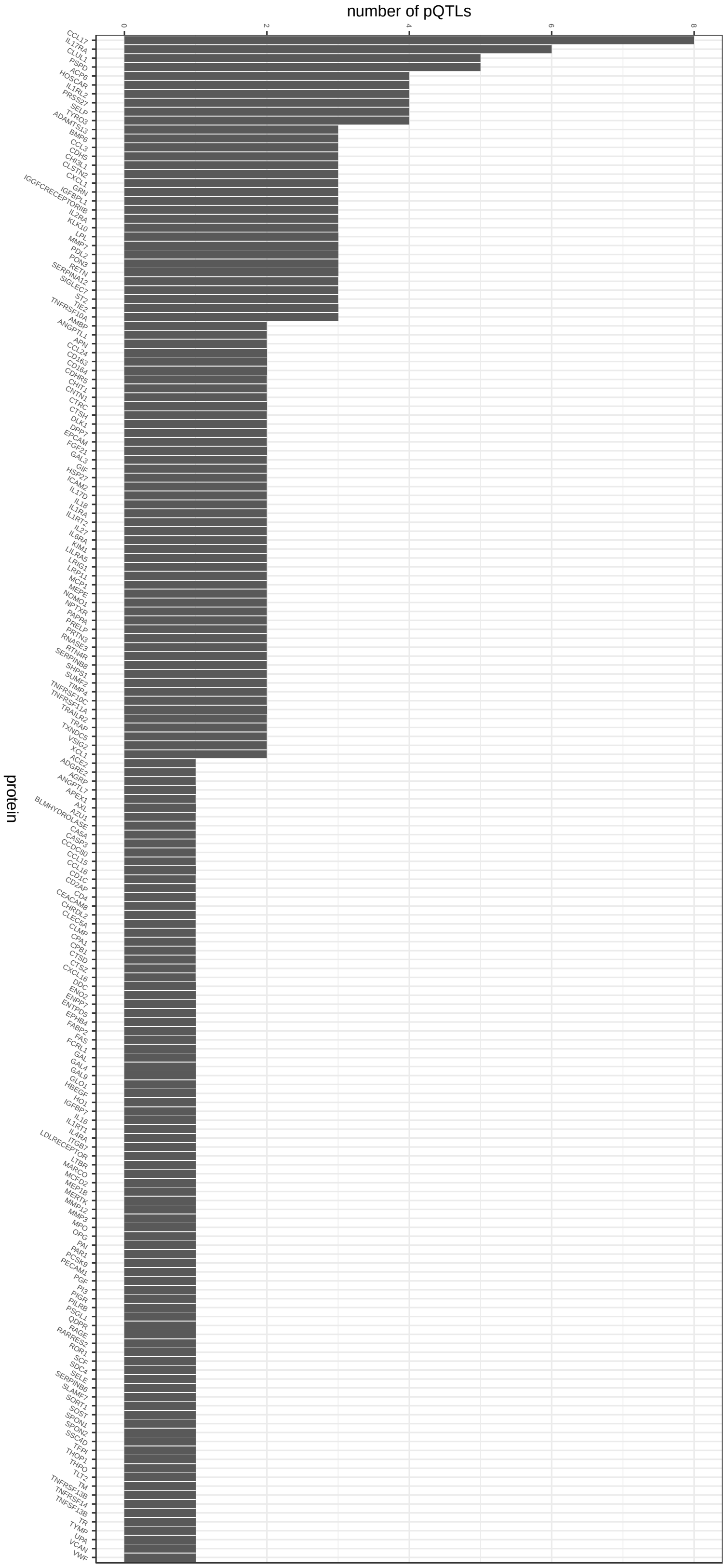

Supplementary Figure 1. Number of conditionally-independent pQTL variants detected for 171 proteins, using a p-value significance threshold of ($7.45x10^{-11}$). No pQTLs were detected for another 84 (33%) proteins included in the analysis.

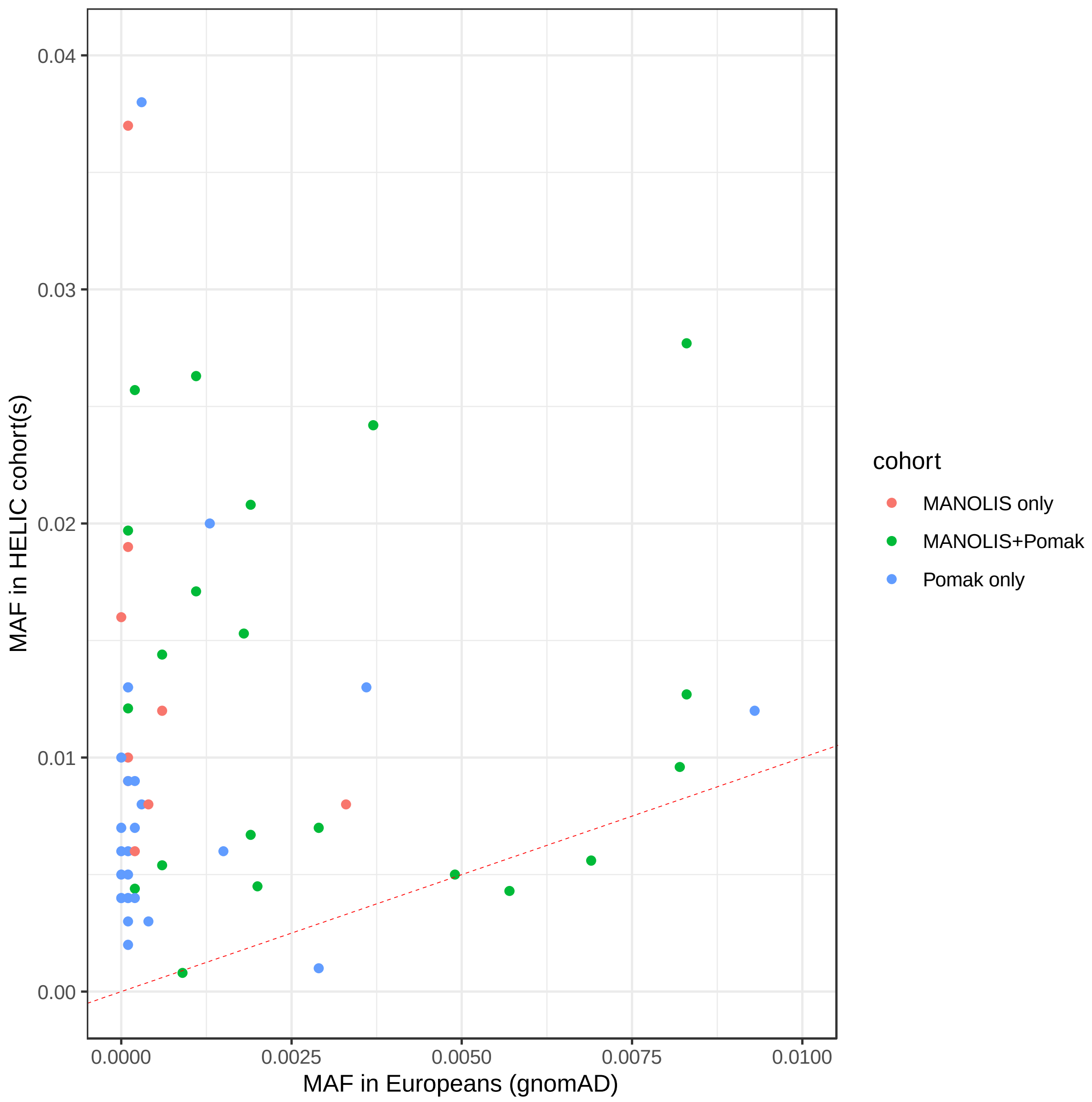

Supplementary Figure 2. Rare (minor allele frequency [MAF] <1%) pQTL variants are drifted up in frequency in MANOLIS and/or Pomak (y-axis), compared to cosmopolitan European populations (x-axis). The plot includes only variants for which a corresponding minor allele frequency in gnomAD non-Finnish Europeans is available. This also excludes variants that are non-existent (MAF=0) in gnomAD non-Finnish Europeans.

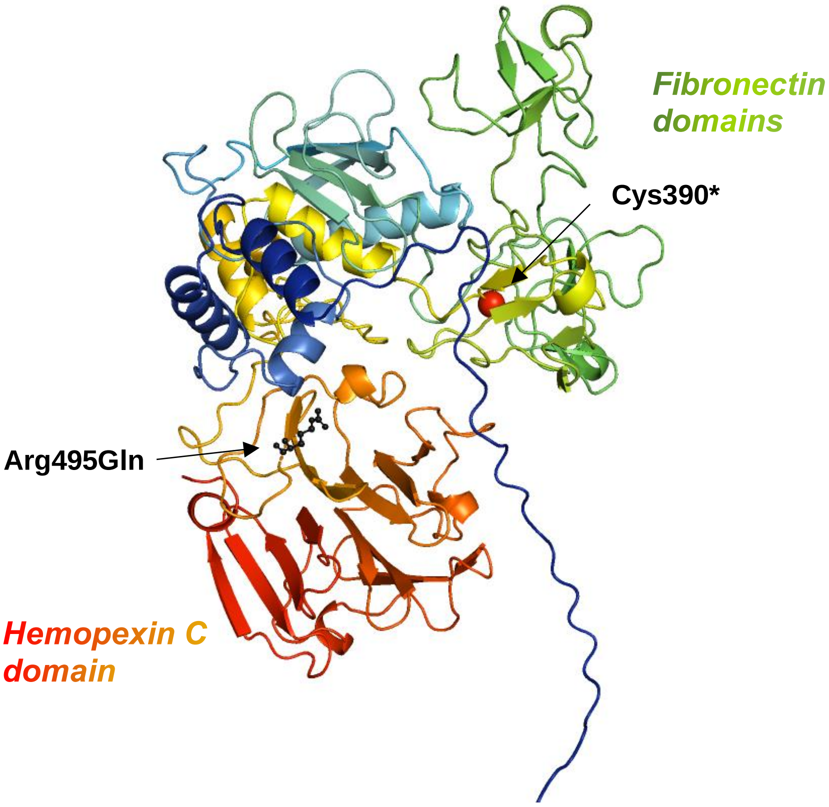

Supplementary Figure 3. 3D structure of 72 kDa type IV collagenase (MMP2). The missense pQTL rs144755357 (Arg495Gln) is represented by a black ball-and-stick model and is located within the hemopexin C domain; another novel Pomak-exclusive stop-gain pQTL 16:55489814 (Cys390*) is represented by a red sphere and is located within a fibronectin domain.

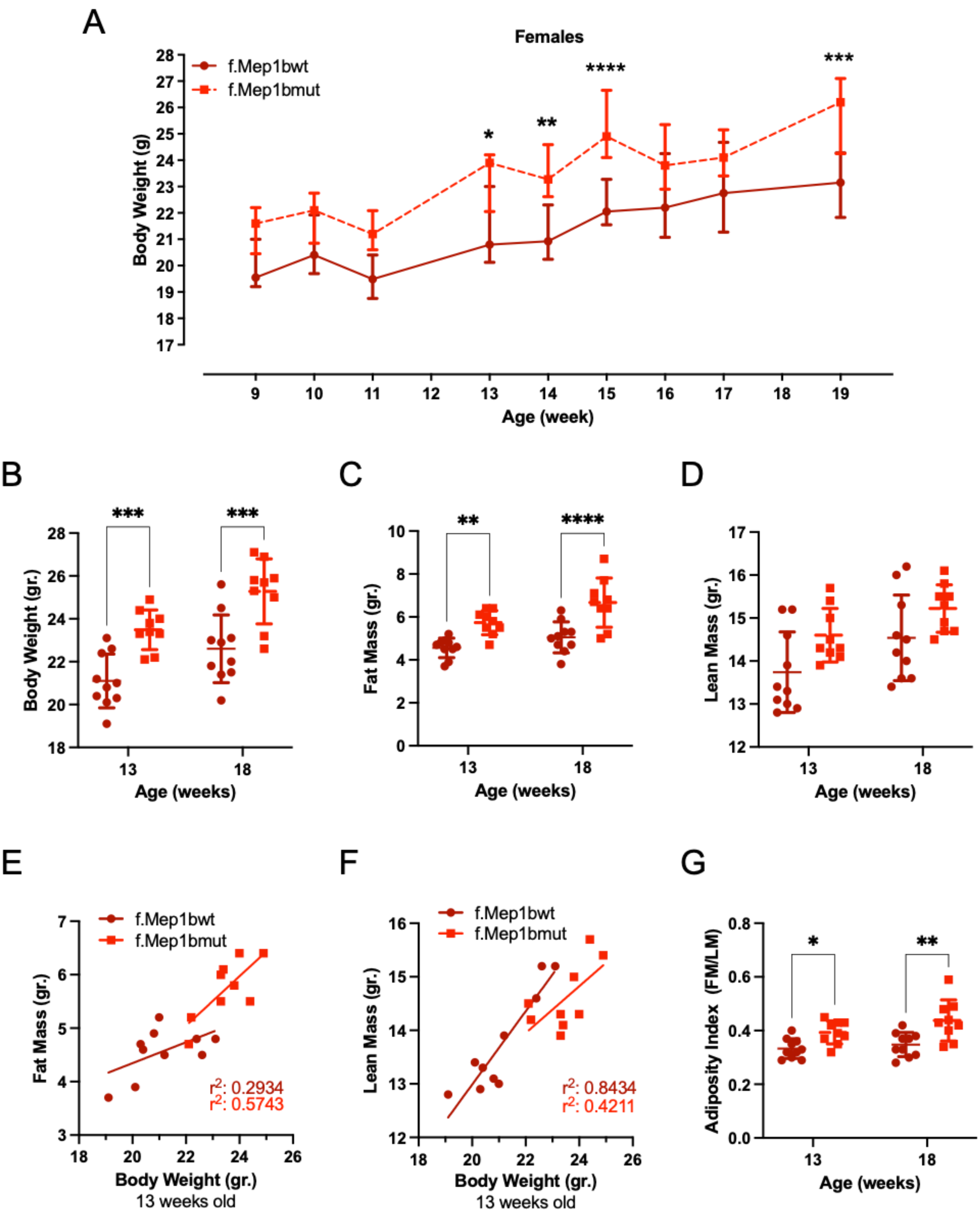

Supplementary Figure 4. Phenotype comparison between female Mep1b KO mice (f.Mep1bmut) and female control mice (f.Mep1bwt). (a) Body weight curve of female control (f.Mep1bwt) and Mep1b KO (f.Mep1bmut) mice from 9 to 19 weeks of age indicated in grams. n= 9-10 per sex and genotype, Median and Interquartile range. Significant genotype:age interaction, mutant female mice have subtle higher body weight that increase with age (p= 0.002). (b) Body weight indicated in grams at the age of 13 and 18 weeks measured before qNMR (two-way ANOVA, multiple comparison). (c) Fat mass expressed in grams per mouse at the age of 13 and 18 weeks (two-way ANOVA, multiple comparison). (d) Lean mass indicated in grams per mouse at the age of 13 and 18 weeks (two-way ANOVA, multiple comparison). (e) Fat mass and body weight correlation at 13 weeks of age. (f) Lean mass and body weight correlation at 13 weeks of age. (g) Adiposity index intended as ratio between fat and lean mass at 13 and 18 weeks of age (two-way ANOVA, multiple comparison); n= 10 control (f.Mep1bwt) and 9

Mep1b KO (f.Mep1bmut) females. Data are shown as mean ± SD. * P ⩽ 0.05, ** P ⩽ 0.01, *** P ⩽ 0.001, **** P ⩽ 0.0001.

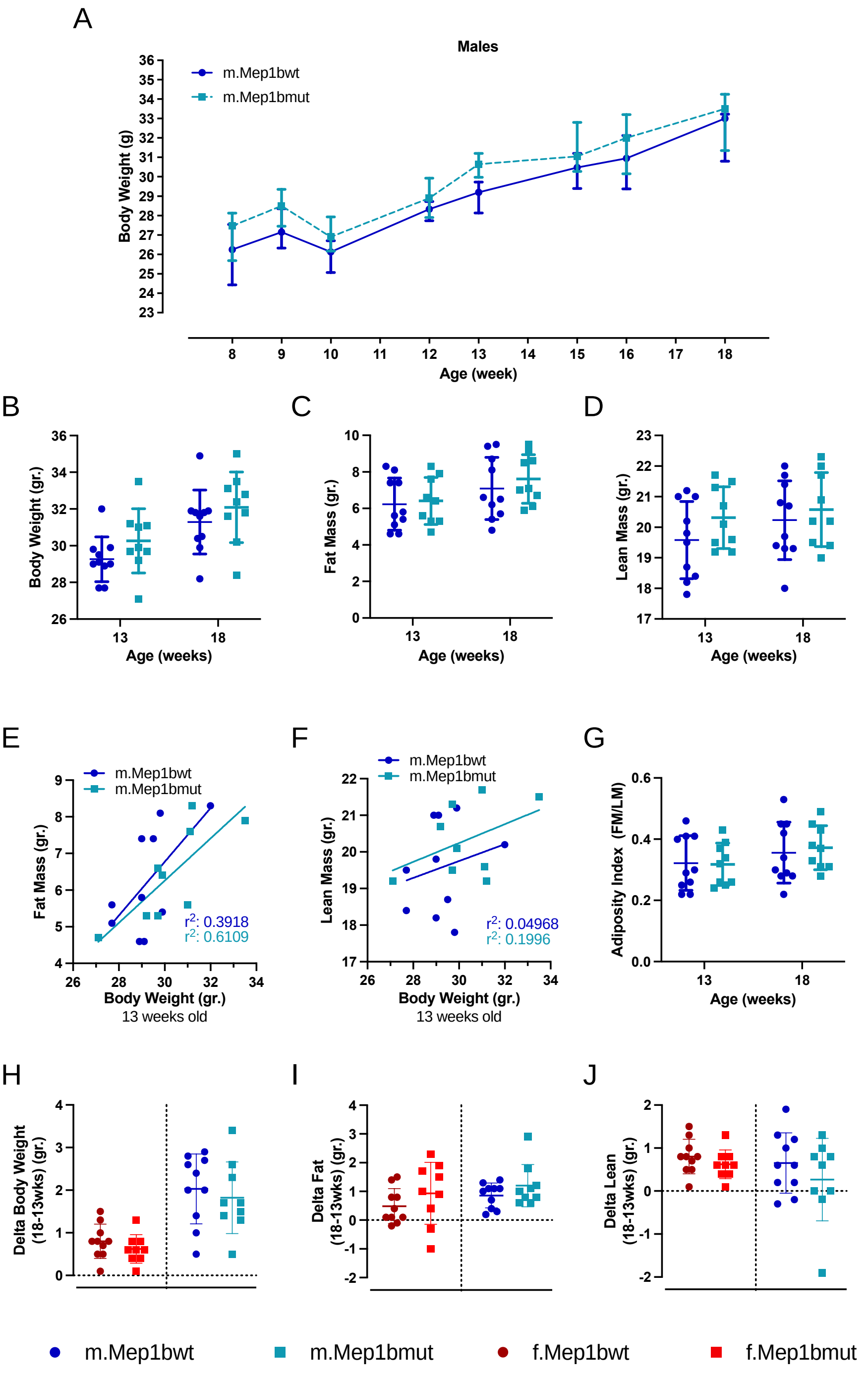

Supplementary Figure 5. Phenotype comparison between male control (m.Mep1bwt) and male Mep1b KO mice (m.Mep1bmut). (a) Body weight curve of male control (m.Mep1bwt) and Mep1b KO (m.Mep1bmut) mice from 8 to 18 weeks of age indicated in grams. n=9-10 per sex and genotype, Median and Interquartile range. Significant genotype:age interaction, mutant female mice have subtle higher body weight that increase with age (p=0.002). (b) Body weight indicated in grams at the age of 13 and 18, when the body composition was also quantified (two-way ANOVA, multiple comparison). (c) Fat mass indicated in grams per mouse at the age of 13 and 18 weeks (two-way ANOVA, multiple comparison). (d) Lean mass indicated in grams per mouse at the age of 13 and 18 weeks (two-way

ANOVA, multiple comparison). (e) Fat mass and Body weight correlation at 13 weeks of age. (f) Lean mass and body weight correlation at 13 weeks of age. (g) Adiposity index intended as ratio between fat and lean mass at 13 and 18 weeks of age. (h) Delta body in grams per mouse, indicating the body mass increment between 13 and 18 weeks of age (two-way ANOVA, multiple comparison). (i) Delta fat amount in grams per mouse, indicating fat increment or reduction between 13 and 18 weeks of age (two-way ANOVA, multiple comparison). (j) Delta lean amount in grans per mouse, indicating lean mass increment or reduction between 13 and 18 weeks of age (two-way ANOVA, multiple comparison). N=10 control (m.Mep1bwt) and 9 Mep1b KO (m.Mep1bmut) males. Data are shown as mean ± SD.

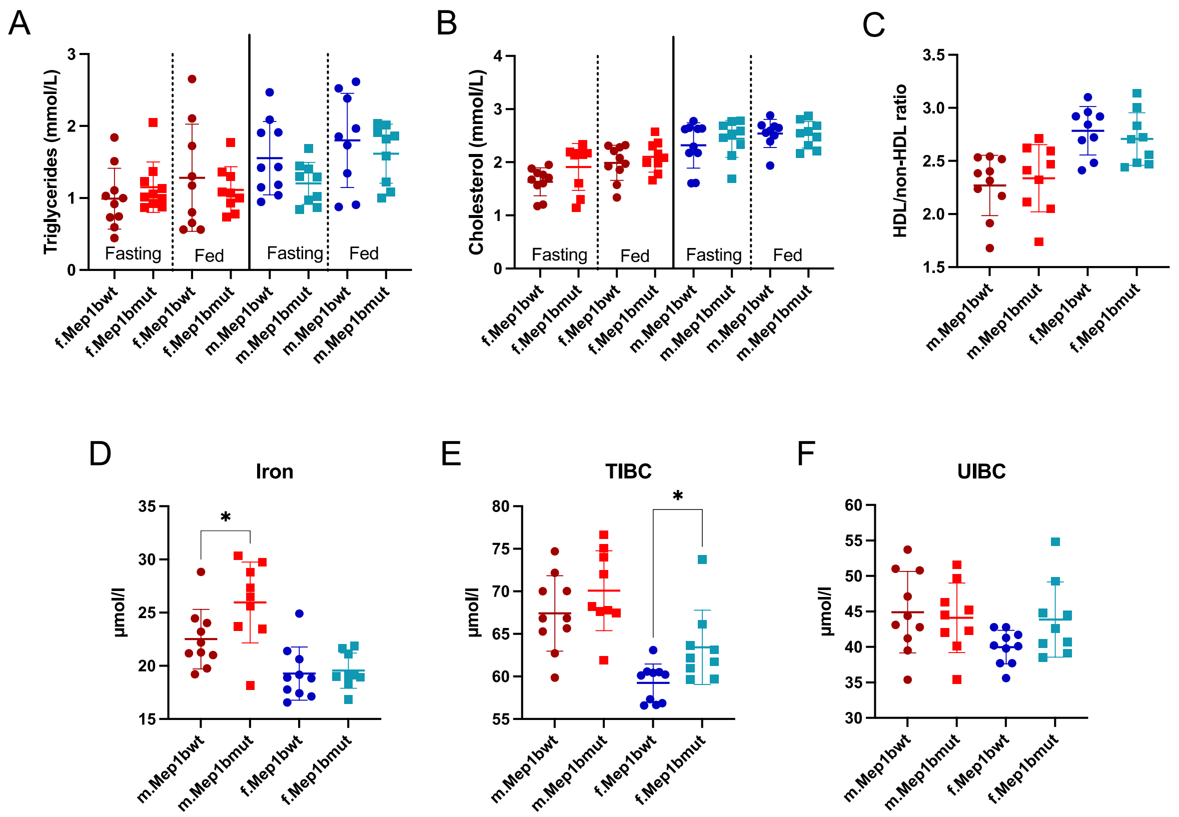

Supplementary Figure 6. Plasma lipid levels and iron metabolism associated parameters in Mep1b knockout mice and wild-type littermates: Triglyceride (a) and total cholesterol (b) levels in overnight fasted (11-12 weeks) and ad libitum fed (19-20 weeks). HDL-cholesterol /non-HDL-cholesterol ratio in overnight fasted animals (c), plasma iron concentration (d) and TIBC calculated from iron UIBC (f). (two-way ANOVA, multiple comparison). Data are shown as mean ± SD. * P ≤ 0.05.

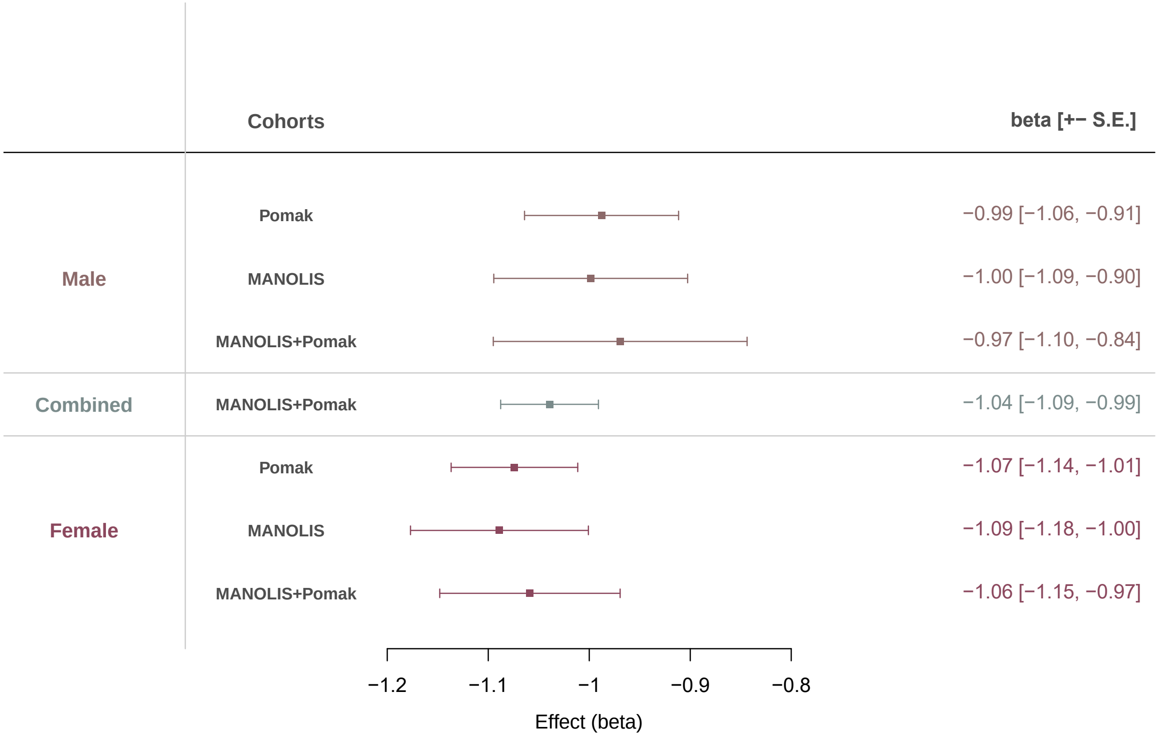

Supplementary Figure 7. Differences in the effect (beta values) of the MEP1B pQTL rs680321-C between males and females. Sex heterogeneity p-value=0.086. ‘MANOLIS+Pomak’ refers to the effect obtained in the meta-analysis between MANOLIS and Pomak.

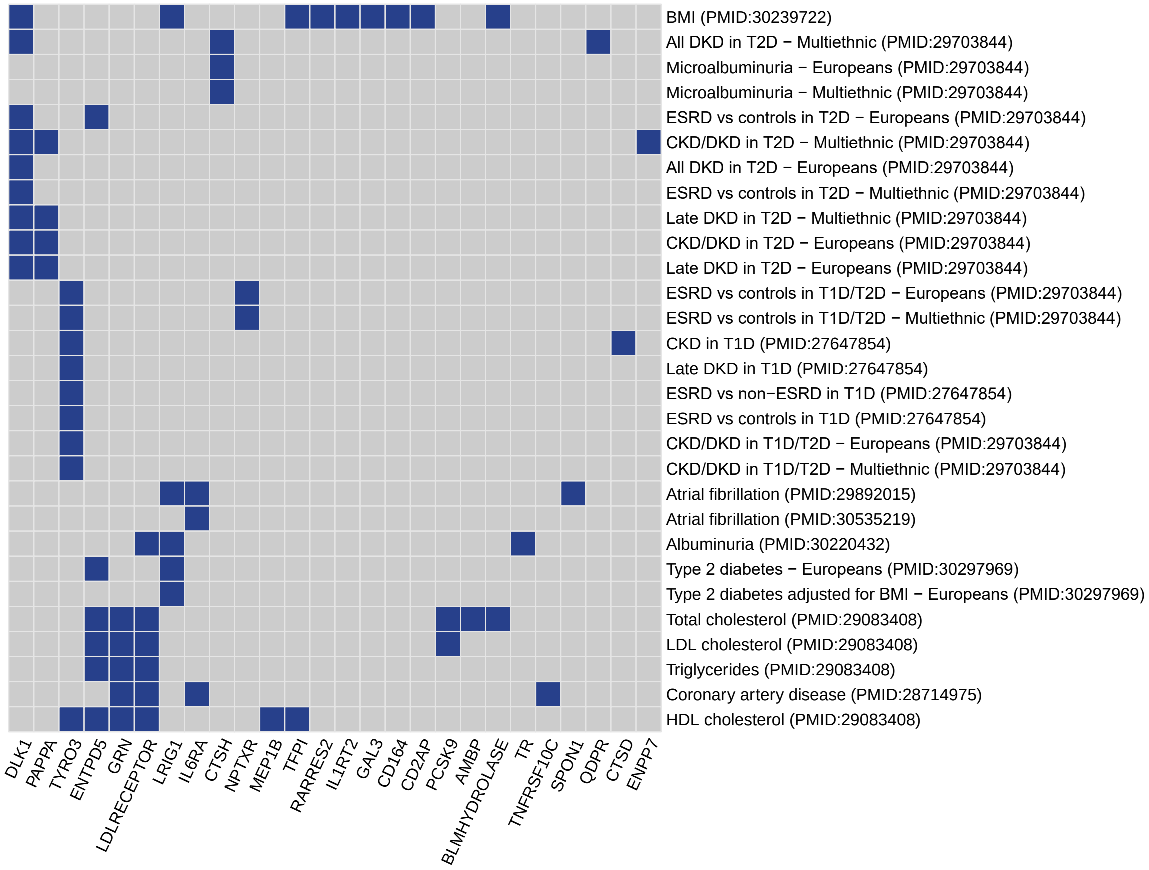

Supplementary Figure 8. Clustering of cardiometabolic traits (y-axis) that share common causal proteins (x-axis), according to two-sample Mendelian randomisation (Wald ratio or inverse-variance weighted). Protein-trait pairs that are significantly associated (FDR-adjusted P<0.05) are represented by blue squares.

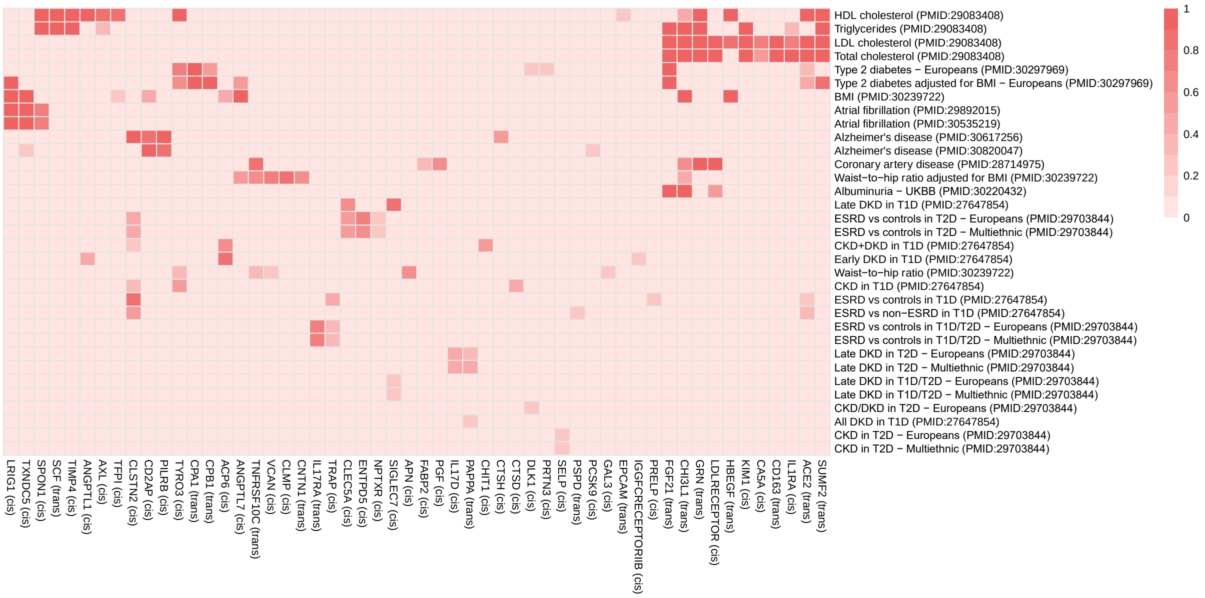

Supplementary Figure 9. Clustering of cardiometabolic traits (y-axis) that share a causal variant with serum proteins (x-axis), according to genetic colocalisation analysis. Squares are coloured according to their colocalisation posterior probability (pp4) as in the legend.

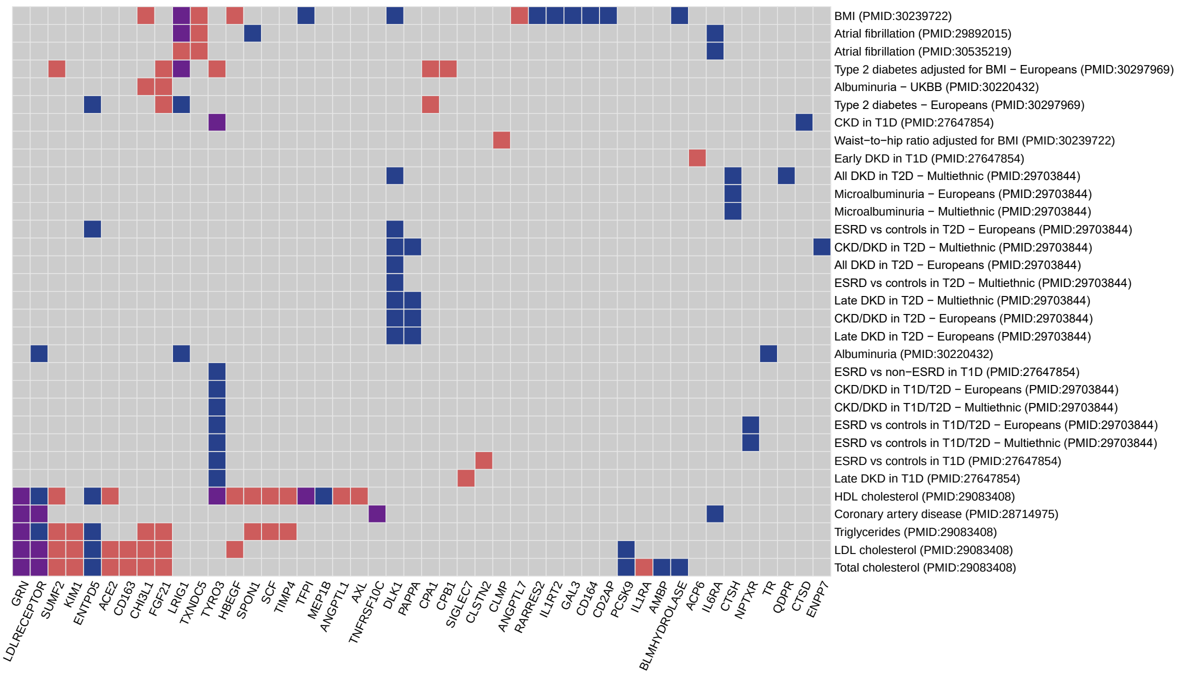

Supplementary Figure 10. Clustering of cardiometabolic traits associated with shared proteins. For each given cardiometabolic trait (y-axis), proteins (x-axis) that are causally associated with the trait according to two-sample Mendelian randomisation (using either of the Wald ratio or inverse-variance weighted methods and both cis and trans pQTLs as instrumental variables) are represented by a blue box; proteins that share a causal variant (either cis or trans pQTLs) are represented by a red box; and proteins with both MR and colocalisation evidence are represented in purple. BMI: body mass index; T2D: type 2 diabetes; CAD: coronary artery disease; T1D: type 1 diabetes; CKD: chronic kidney disease; DKD: diabetic kidney disease; ESRD: end-stage renal disease; HDL: high-density lipoprotein; LDL: low-density lipoprotein.

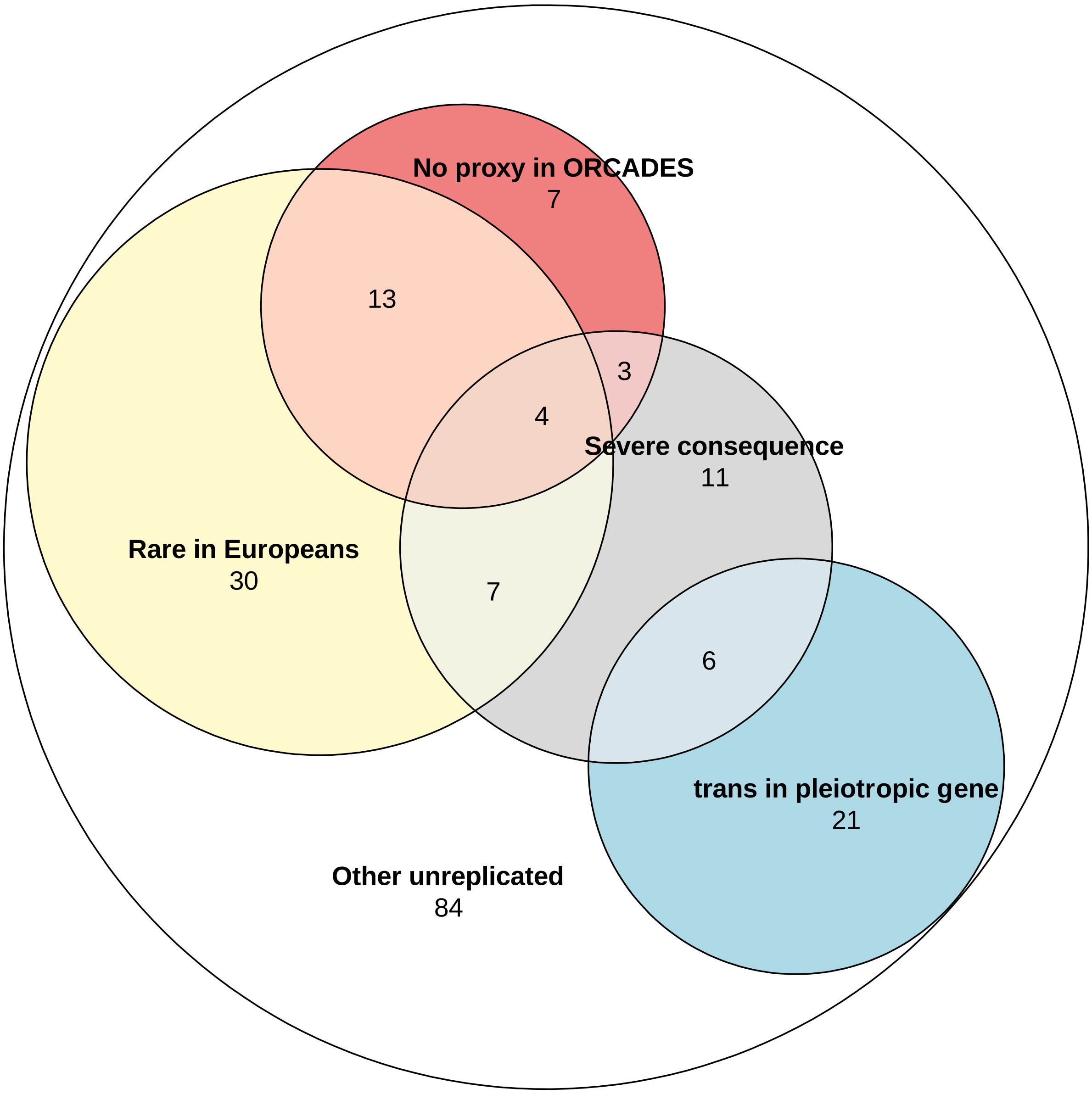

Supplementary Figure 11. Characterisation of 186 pQTL variants present in at least one discovery cohort that could not (due to lack of LD proxies) or failed to replicate in the ORCADES cohort.

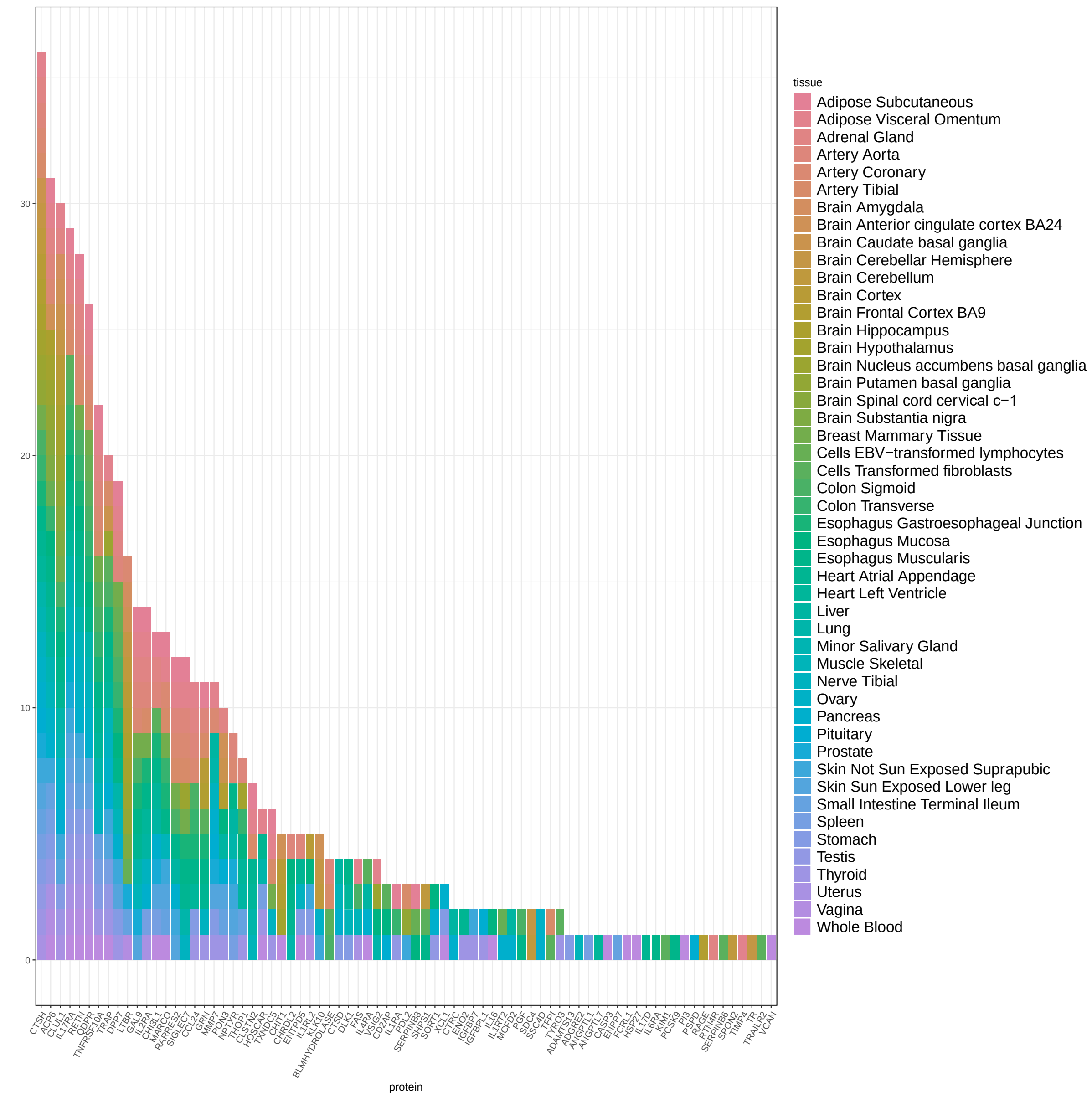

Supplementary Figure 12. Cis-pQTLs for 77 proteins colocalise with eQTLs for their encoding target gene in multiple tissues. For every protein, all cis-pQTLs were tested. Positive colocalisation was defined as a colocalisation posterior probability (PP4) of >80%.

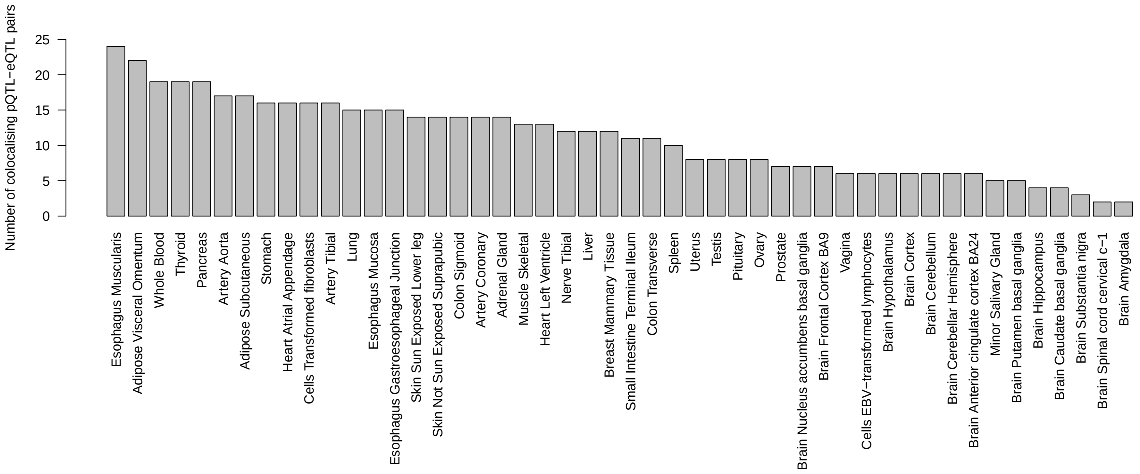

Supplementary Figure 13. Ranking tissues according to the number of colocalising pQTL-eQTL pairs.
