# Supplementary material for: Identifying causal serum protein–cardiometabolic trait relationships using whole genome sequencing
Source: Hum Mol Genet. 2022 Nov 9;32(8):1266–75. doi: 10.1093/hmg/ddac275 (PMC10077504; doi:10.1093/hmg/ddac275)
Supplement: GP_Supplementary-text_ddac275 [file gp_supplementary-text_ddac275.pdf]

Supplementary text

Supplementary Note 1

Colocalisation with gene expression QTLs

For *cis*-pQTLs, colocalisation with a gene expression QTL (eQTL) in a particular tissue is an indicator of shared genetic architecture and transcriptional regulation, and can provide a relative comparison of the range of tissue expression of proteins (Supplementary Figure 12 and 13). We observe 77 (55%) *cis*-pQTLs colocalising with eQTLs in at least one tissue, similar to a recent study(1). Two *cis*-pQTLs for CTSH colocalised with eQTLs in the greatest number of tissues (36 out of 48 tested), indicating expression of the protein in a wide range of tissues. We find however that the two pQTLs (rs62013200 and rs2289702) colocalised with eQTLs in only eight common tissues, suggesting distinct regulatory mechanisms between tissues and emphasising a demand for future tissue and cell-type specific pQTL analyses to deepen our understanding of the genetic regulation of proteins.

For *trans*-pQTLs, colocalisation with eQTLs is one method used to map them to their causal genes. Sixty-one (72.6%) *trans*-pQTLs colocalised with an eQTL for at least one nearby gene in any tissue. We previously showed that testing a 2Mb region around the pQTL and using a stringent threshold for positive colocalisation (PP4>0.8) could detect 71% of causal genes for *cis*-pQTLs(2), and we expect that this number will be lower for *trans*-pQTLs due to reduced power as a result of smaller effect sizes. Future work will include combining eQTL colocalisation with additional methods such as literature mining and pathway analysis to confidently map causal *trans* genes.

Supplementary Note 2

Detailed description of more novel protein-cardiometabolic trait associations

Through two-sample MR, we recapitulate robust and established protein-disease causal associations for LDLR and lipid levels; LRIG1 and atrial fibrillation; IL6RA (interleukin-6 receptor subunit alpha precursor) and coronary artery disease(3); LPL (lipoprotein lipase) and lipid levels(4,5); and MMP12 (matrix metalloproteinase-12) and ischaemic stroke(6,7).

Increased TYRO3 and CTSH levels are associated with an increased risk of DKD in individuals with type 1 or 2 diabetes, and reduced DLK1 levels are associated with an increased risk of DKD in individuals with T2D. CTSH is a cysteine protease of the family of cathepsins, whose role in kidney disease has been extensively studied(8), although CTSH specifically has not been associated with kidney disease. DLK1 is an inhibitor of Notch signalling(9), which is a pathway involved in numerous biological processes. Variation in the *DLK1* gene has been associated in previous studies with T2D(10) and glycated haemoglobin (HbA1c) levels(11), a biomarker for diabetes, while knockout mice have decreased lean body mass and circulating glucose and increased lean body mass(12).

A newly-identified *trans*-pQTL for serum SUMF2 (rs1461729; MAF=0.068; beta=0.373; SE=0.054; P=3.12x10^{-12}) is associated with increased levels of the protein. Two-sample MR results suggest that the protein is causally associated with decreased HDL, LDL, and total cholesterol; and with increased fasting insulin, fasting glucose, and type 2 diabetes (T2D) risk. This is further supported by positive colocalisation between the *trans*-pQTL and genetic association signals for all of the above traits (Supplementary Table 7). The pQTL is located at chr8q23.1, and is in linkage disequilibrium (r^2=0.77) with a known T2D-associated variant, rs4841132 (MAF=0.062; beta=0.368; SE=0.056; P=3.99x10^{-11}). Both variants reside within a long non-coding RNA (lncRNA) transcript, *LOC157273*, which regulates hepatic glycogen deposition and the expression of a large number of genes(13). Another novel *cis*-pQTL for SUMF2 is associated with decreased serum SUMF2

(opposite effect from the *trans*-pQTL), but shows no evidence of causality for the same traits.

We also report a causal association between decreased TNFRSF10C (decoy receptor 1 or TRAIL-R3) and increased risk of coronary artery disease (CAD) (beta=-0.045; SE= 0.011; PFDR=4.20x10-5). The MR association is driven by one cis¬-pQTL and one trans-pQTL; the trans-pQTL (rs4760; MAF=0.154; P=1.06x10-266; beta=-1.12; SE=0.032) is a known6,10 missense variant located within the PLAUR gene, which is associated with decreased serum TNFRSF10C and also colocalises with signals for CAD (colocalisation posterior probability [PP]=89.6%) and other blood cell traits (Supplementary Table 7). PLAUR encodes the urokinase receptor (uPAR); uPAR and its ligand, uPA, were not significantly associated with rs4760 in our analysis. We find that the trans-pQTL colocalises very strongly (PP=98.2%) with a gene expression QTL (eQTL) for CADM4 (cell adhesion molecule 4), suggesting that the variant may influence TNFRSF10C levels through CADM4. Pathway analysis using the STRING database (https://string-db.org/) showed no direct interactions between the two proteins. Further experiments are required to confidently map the target gene.

Supplementary Note 3

Description of the genetic architecture of serum MEP1B

Rs680321 is in LD (r^2>0.8) with one missense variant (rs616114; MAF=0.40) and one splice region variant (rs335518; MAF=0.44). For the missense rs616114, the amino acid replacement (P695L) might lead to altered phosphorylation in close proximity to the C-terminus of MEP1B, which could impact the serum turnover of the protein(14). Rs616114 is also associated with expression of *MEP1B* in lung tissue (GTEx), suggesting protein regulation at the transcriptional level.

Supplementary Note 4

Characterisation and discussion of the Mep1b KO mouse

We carried out a target disruption of the catalytic centre of MEP1B, caused by a deletion of exon 7 of the wild-type allele interrupted by a neomycin resistance gene, resulting in a full body KO mouse model. We have previously shown that Mep1B absence does not result in embryonic lethality nor overtly altered phenotypes; however, evidence suggests that it leads to changes in kidney gene expression(15,16).

At the German Mouse Clinic, we systematically phenotyped *Mep1B* KO animals generated from heterozygous crossings. Monitoring body weight from age 9 to 19 weeks, we observed a constantly increased body mass over time in female mutants compared to controls (Supplementary Figure 4A), while males showed a similar, but not significant trend (Supplementary Figure 5A). Body composition analysis at two time points revealed that significantly higher body weight in female mutants was mainly consequent to an increase in fat mass (Supplementary Figure 4B; C; Supplementary Table 8A-B). Lean mass was also slightly higher (Supplementary Figure 4D) but did not reach statistical significance. In mutant females, fat mass correlated better than lean mass with body weight; whereas, for control females, we observed the opposite (Supplementary Figure 4E; F). The higher fat/lean mass ratio (adiposity index) further highlights higher fat content over remaining lean body mass in mutant females compared to control females (Supplementary Figure 4G). Male mutants did not show a clear genotype-dependent shift in body composition (Supplementary Figure 5B-G). Finally, both mutant male and female mice gained similar body mass, fat, and lean mass amounts between the two measurements (13 and 18 weeks of age) compared to the controls (Supplementary Figure 5H-J), suggesting that changes in body composition stem from earlier age. As only female knockout mice were affected, we also investigated the sex-specific effect of the MEP1B pQTL, where we note a slightly stronger, albeit non-significant effect in females (Supplementary Figure 7).

Although an increase in HDL cholesterol levels has been found to be associated with decreased MEP1B protein concentrations in humans, we did not detect significant effects

of the *Mep1b* knockout on plasma triglyceride or cholesterol levels in overnight fasted or ad libitum fed state mice (Supplementary Figure 6A, B). The ratio of HDL and non-HDL cholesterol was also not significantly altered in mutant mice (Supplementary Figure 6C). *Mep1b* KO mice showed subtle alteration in iron metabolism-related parameters; namely, elevated plasma iron concentration and calculated total iron binding capacity (TIBC), while unsaturated plasma iron binding capacity (UIBC) was comparable for mutant and control mice (Supplementary Figure 6D-F). Increased renal transferrin receptor expression has also been observed in a previous study(16). Increased TIBC −a surrogate marker of transferrin levels− hints towards increased hepatic transferrin production, usually upregulated in response to intracellular iron deficiency, while ferritin production is downregulated under this condition(17). An association of cellular iron metabolism with the regulation of glucose metabolism and type 2 diabetes has been recently described(18,19).

Lipoprotein profiles in mice compared to humans show distinct differences. While low density lipoproteins comprise the major lipoprotein fraction in humans, HDL is the dominating lipoprotein in mice. The general composition of VLDL, LDL, and HDL lipoprotein fractions is similar in mice and humans(20); however, HDL can be further divided into subfractions in both mouse and man, with considerable differences in composition between species(21). These differences might account for the fact that no clear alteration of plasma HDL levels was observed in *Mep1b* KO mice. Indeed, an alteration of only one subfraction may be obscured by other unaffected portions of the HDL lipoprotein fraction. Further studies are required to analyse this in detail.

Supplementary references

1. Pietzner, M., Wheeler, E., Carrasco-Zanini, J., Cortes, A., Koprulu, M., Wörheide, M.A., Oerton, E., Cook, J., Stewart, I.D., Kerrison, N.D., *et al.* (2021) Mapping the proteo-genomic convergence of human diseases. *Science*, **374**, eabj1541.
2. Png, G., Barysenka, A., Repetto, L., Navarro, P., Shen, X., Pietzner, M., Wheeler, E., Wareham, N.J., Langenberg, C., Tsafantakis, E., *et al.* (2021) Mapping the serum proteome to neurological diseases using whole genome sequencing. *Nat. Commun.*, **12**, 7042.
3. Lim, G.B. (2012) Coronary artery disease: IL-6 signaling linked with CHD. *Nat. Rev. Cardiol.*, **9**, 313.
4. Merkel, M., Eckel, R.H. and Goldberg, I.J. (2002) Lipoprotein lipase: genetics, lipid uptake, and regulation. *J. Lipid Res.*, **43**, 1997–2006.
5. Basu, D. and Goldberg, I.J. (2020) Regulation of lipoprotein lipase-mediated lipolysis of triglycerides. *Curr. Opin. Lipidol.*, **31**, 154–160.
6. Traylor, M., Mäkelä, K.-M., Kilarski, L.L., Holliday, E.G., Devan, W.J., Nalls, M.A., Wiggins, K.L., Zhao, W., Cheng, Y.-C., Achterberg, S., *et al.* (2014) A novel MMP12 locus is associated with large artery atherosclerotic stroke using a genome-wide age-at-onset informed approach. *PLoS Genet.*, **10**, e1004469.
7. Mahdessian, H., Perisic Matic, L., Lengquist, M., Gertow, K., Sennblad, B., Baldassarre, D., Veglia, F., Humphries, S.E., Rauramaa, R., de Faire, U., *et al.* (2017) Integrative studies implicate matrix metalloproteinase-12 as a culprit gene for large-artery atherosclerotic stroke. *J. Intern. Med.*, **282**, 429–444.
8. Cocchiaro, P., De Pasquale, V., Della Morte, R., Tafuri, S., Avallone, L., Pizard, A., Moles, A. and Pavone, L.M. (2017) The Multifaceted Role of the Lysosomal Protease Cathepsins in Kidney Disease. *Front. Cell Dev. Biol.*, **5**, 114.
9. Nueda, M.-L., González-Gómez, M.-J., Rodríguez-Cano, M.-M., Monsalve, E.-M., Díaz-Guerra, M.J.M., Sánchez-Solana, B., Laborda, J. and Baladrón, V. (2018) DLK proteins modulate NOTCH signaling to influence a brown or white 3T3-L1 adipocyte fate. *Sci. Rep.*, **8**, 16923.
10. Parra, E.J., Below, J.E., Krithika, S., Valladares, A., Barta, J.L., Cox, N.J., Hanis, C.L., Wacher, N., Garcia-Mena, J., Hu, P., *et al.* (2011) Genome-wide association study of type 2 diabetes in a sample from Mexico City and a meta-analysis of a Mexican-American sample from Starr County, Texas. *Diabetologia*, **54**, 2038–2046.
11. Sinnott-Armstrong, N., Tanigawa, Y., Amar, D., Mars, N., Benner, C., Aguirre, M., Venkataraman, G.R., Wainberg, M., Ollila, H.M., Kiiskinen, T., *et al.* (2021) Genetics of 35 blood and urine biomarkers in the UK Biobank. *Nat. Genet.*, **53**, 185–194.
12. The International Mouse Phenotyping Consortium, Dickinson, M.E., Flenniken, A.M., Ji, X., Teboul, L., Wong, M.D., White, J.K., Meehan, T.F., Weninger, W.J., Westerberg, H.,

et al. (2016) High-throughput discovery of novel developmental phenotypes. *Nature*, **537**, 508–514.

13. Manning, A.K., Goustin, A.S., Kleinbrink, E.L., Thepsuwan, P., Cai, J., Ju, D., Leong, A., Udler, M.S., Brown, J.B., Goodarzi, M.O., *et al.* (2020) A Long Non-coding RNA, LOC157273, Is an Effector Transcript at the Chromosome 8p23.1-PPP1R3B Metabolic Traits and Type 2 Diabetes Risk Locus. *Front. Genet.*, **11**, 615.
14. Armbrust, F., Bickenbach, K., Koudelka, T., Tholey, A., Pietrzik, C. and Becker-Pauly, C. (2021) Phosphorylation of meprin β controls its cell surface abundance and subsequently diminishes ectodomain shedding. *FASEB J. Off. Publ. Fed. Am. Soc. Exp. Biol.*, **35**, e21677.
15. Sterchi, E.E., Stöcker, W. and Bond, J.S. (2008) Meprins, membrane-bound and secreted astacin metalloproteinases. *Mol. Aspects Med.*, **29**, 309–328.
16. Norman, L.P., Jiang, W., Han, X., Saunders, T.L. and Bond, J.S. (2003) Targeted disruption of the meprin beta gene in mice leads to underrepresentation of knockout mice and changes in renal gene expression profiles. *Mol. Cell. Biol.*, **23**, 1221–1230.
17. Muckenthaler, M.U., Galy, B. and Hentze, M.W. (2008) Systemic iron homeostasis and the iron-responsive element/iron-regulatory protein (IRE/IRP) regulatory network. *Annu. Rev. Nutr.*, **28**, 197–213.
18. Santos, M.C.F.D., Anderson, C.P., Neschen, S., Zumbrennen-Bullough, K.B., Romney, S.J., Kahle-Stephan, M., Rathkolb, B., Gailus-Durner, V., Fuchs, H., Wolf, E., *et al.* (2020) Irp2 regulates insulin production through iron-mediated Cdkal1-catalyzed tRNA modification. *Nat. Commun.*, **11**, 296.
19. Liu, J., Li, Q., Yang, Y. and Ma, L. (2020) Iron metabolism and type 2 diabetes mellitus: A meta-analysis and systematic review. *J. Diabetes Investig.*, **11**, 946–955.
20. Camus, M.C., Chapman, M.J., Forgez, P. and Laplaud, P.M. (1983) Distribution and characterization of the serum lipoproteins and apoproteins in the mouse, Mus musculus. *J. Lipid Res.*, **24**, 1210–1228.
21. Gordon, S.M., Li, H., Zhu, X., Shah, A.S., Lu, L.J. and Davidson, W.S. (2015) A comparison of the mouse and human lipoproteome: suitability of the mouse model for studies of human lipoproteins. *J. Proteome Res.*, **14**, 2686–2695.
